# Sarcopenia is associated with a greater risk of polypharmacy and number of medications: a systematic review and meta‐analysis

**DOI:** 10.1002/jcsm.13190

**Published:** 2023-02-13

**Authors:** Konstantinos Prokopidis, Panagiotis Giannos, Jean Yves Reginster, Olivier Bruyere, Mirko Petrovic, Antonio Cherubini, Konstantinos K. Triantafyllidis, Konstantinos S. Kechagias, Yannis Dionyssiotis, Matteo Cesari, Kinda Ibrahim, David Scott, Mario Barbagallo, Nicola Veronese, Special interest group in Systematic Reviews and Meta‐analyses and the Task Force on Pharmaceutical Strategy of the European Geriatric Medicine Society (EuGMS)

**Affiliations:** ^1^ Department of Musculoskeletal Biology, Institute of Life Course and Medical Sciences University of Liverpool Liverpool UK; ^2^ Society of Meta‐research and Biomedical Innovation London UK; ^3^ Department of Life Sciences, Faculty of Natural Sciences Imperial College London London UK; ^4^ WHO Collaborating Center for Epidemiology of Musculoskeletal Health and Aging Liège Belgium; ^5^ Division of Public Health, Epidemiology and Health Economics University of Liège Liège Belgium; ^6^ Division of Public Health, Epidemiology and Health Economics, WHO Collaborating Center for Public Health Aspects of Musculo‐Skeletal Health and Ageing University of Liège Liège Belgium; ^7^ Section of Geriatrics, Department of Internal Medicine and Paediatrics Ghent University Ghent Belgium; ^8^ Geriatria, Accettazione Geriatrica e Centro di Ricerca per l'Invecchiamento, IRCCS INRCA Ancona Italy; ^9^ Department of Nutrition and Dietetics Homerton University Hospital Foundation Trust London UK; ^10^ Department of Metabolism, Digestion and Reproduction, Faculty of Medicine Imperial College London London UK; ^11^ Medical School, Spinal Cord Injury Rehabilitation Clinic, General University Hospital Patras University of Patras Patras Greece; ^12^ Department of Clinical Sciences and Community Health University of Milan Milan Italy; ^13^ Geriatric Unit IRCCS Istituti Clinici Scientifici Maugeri Milan Italy; ^14^ Academic Geriatric Medicine, Faculty of Medicine, University Hospital Southampton University of Southampton Southampton UK; ^15^ Applied Research Collaboration Wessex, The National Institute of Health and Care Research (NIHR) University of Southampton Southampton UK; ^16^ Institute for Physical Activity and Nutrition (IPAN), School of Exercise and Nutrition Sciences Deakin University Burwood Victoria Australia; ^17^ Department of Medicine, School of Clinical Sciences at Monash Health Monash University Clayton Victoria Australia; ^18^ Department of Internal Medicine and Geriatrics University of Palermo Palermo Italy

**Keywords:** polypharmacy, medications, sarcopenia, physical function, ageing

## Abstract

Polypharmacy in older adults is associated with multiple negative consequences that may affect muscular function, independently from the presence of medical conditions. The aim of this systematic review and meta‐analysis was to investigate the association of sarcopenia with polypharmacy and higher number of medications. A systematic literature search of observational studies using PubMed, Web of Science, Scopus and Cochrane Library databases was conducted from inception until June 2022. To determine if sarcopenia is associated with a higher risk of polypharmacy and increased number of medications, a meta‐analysis using a random‐effects model was used to calculate the pooled effects (CRD42022337539). Twenty‐nine studies were included in the systematic review and meta‐analysis. Sarcopenia was associated with a higher prevalence of polypharmacy (odds ratio [OR]: 1.65, 95% confidence interval [CI] [1.23, 2.20], *I* ^2^ = 84%, *P* < 0.01) and higher number of medications (mean difference: 1.39, 95% CI [0.59, 2.19], *I* ^2^ = 95%, *P* < 0.01) compared with individuals without sarcopenia. Using meta‐regression, a high variance was observed due to different populations (i.e., community‐dwelling, nursing home residents, inpatients, outpatients) for both outcomes of polypharmacy (*r* = −0.338, SE = 0.1669, 95% CI [−0.67, −0.01], *z* = −2.03, *P* = 0.04) and number of medications (*r* = 0.589, SE = 0.2615, 95% CI [0.08, 1.10], *z* = 2.25, *P* = 0.02). This systematic review and meta‐analysis reported a significantly increased risk of polypharmacy and higher number of medications in people with sarcopenia compared with individuals without this condition. Future research should clarify whether the specificity and number of medications is a direct contributor in accelerating the progression of muscle wasting and dysfunction contributing to sarcopenia in older adults.

## Introduction

Polypharmacy is defined as the use of multiple concurrent medications or the simultaneous long‐term use of different drugs by the same individual.^1^ Despite that a univocal consensus has not yet established regarding the numerical definition of polypharmacy, several studies have reported that the concurrent use of five or more medications is usually sufficient for the definition of this condition.^1^

The prevalence of polypharmacy in older people is extremely wide ranging, due to differences in age, current health condition, health care setting and geographic location.^2^ Epidemiological data suggest that polypharmacy may affect more than one third of older people worldwide,^3^, ^4^, ^5^ making this phenomenon of great relevance in geriatric medicine. It is widely known that polypharmacy in older populations has been associated with several negative healthcare outcomes, independent from the presence of medical conditions. For example, a large umbrella review found that, among 26 meta‐analyses, polypharmacy was associated with multiple detrimental outcomes, including adverse drug reactions, adverse drug events, disability and hospitalizations.^6^ Interestingly, frailty is associated with less polypharmacy and higher prevalence of symptomatic drugs (i.e., laxatives, paracetamol and opioids) use among nursing home residents compared with non‐frail individuals that were prescribed primarily preventive drugs such as bisphosphonates and acetylsalicylic acid.^7^ However, there is a growing concern about the clinical management of comorbidities and the impact of polypharmacy that could result in potentially inappropriate prescribing.^8^, ^9^, ^10^

There has been an increasing interest in understanding the association between polypharmacy and sarcopenia—the pathological loss of muscle mass, strength and function in older people.^11^ Sarcopenia is associated with an increased risk of impaired physical function,^12^ hospitalization and mortality.^13^ Polypharmacy, particularly specific drugs such as corticosteroids,^14^ has shown to be associated with muscle weakness and low appendicular lean mass in older age.^15^, ^16^ On the other hand, there is some evidence that changes in body weight and composition as well as protein synthesis affect drug distribution and metabolism.^17^ A recent scoping review of the literature found an association between sarcopenia and risk of sarcopenia and polypharmacy or the number of medications in community‐dwelling older people, but not among residents of nursing homes or hospital inpatients.^9^ The results of that review were based on cross‐sectional data, identifying only an association rather than a causal relationship between sarcopenia and polypharmacy or number of medications. Nevertheless, the different existing definitions of sarcopenia and polypharmacy, the wide‐ranging methods of sarcopenia assessment, and the health care setting of older populations have hindered the appropriate evaluation of the relationship between polypharmacy and sarcopenia.^9^ Specifically, antidiabetic drugs may induce transcription factors of myostatin^18^ and blunt hypertrophic responses following exercise,^19^ while beta‐blockers could impair muscular adaptation to exercise, reducing endurance exercise capacity.^20^ In addition, glucocorticoids and anti‐proliferative drugs for cancer treatment could upregulate E3 ubiquitin ligases such as atrogin1, MuRF1, and MUSA1^14^ and reduce the expression of mediators involved in mitochondrial function.^21^ Finally, considering the association between polypharmacy and malnutrition^22^, ^23^ due to potential changes in gastrointestinal microenvironment and the gut microbiota,^24^ deprescribing, that is, judiciously decreasing or stopping a number of medications, may improve nutritional status,^25^ a key contributor in improving sarcopenia status.

There is currently no systematic review and meta‐analysis exploring the quantitative differences regarding the prevalence of polypharmacy and number of medications between older adults with sarcopenia compared with those without sarcopenia. Therefore, in this meta‐analysis, we aimed to investigate the extent at which sarcopenia may amplify the risk of polypharmacy and be associated with higher number of medications. Our analyses might shed light on how multiple variables, such as different definitions of sarcopenia and polypharmacy, body composition assessment tools, geographical location, study population, health status and different age groups among older populations, could impact a polypharmacy/sarcopenia relationship.

## Methods

This systematic review and meta‐analysis was conducted in accordance with the updated 2020 Preferred Reporting Items for Systematic Reviews and Meta‐Analyses (PRISMA) guidelines.^26^ The protocol has been registered in the International Prospective Register of Systematic Reviews (PROSPERO) (CRD: 42022337539).

### Search strategy

Two independent reviewers (K. P. and K. K. T.) searched PubMed, Scopus, Web of Science and Cochrane Library from inception until June 2022. The full search strategy and the search terms used are described in the Supporting information, *Table* *S1*). The searches were re‐run before submission to retrieve any additional studies that met our inclusion criteria. Discrepancies in the literature search process were resolved by a third and fourth investigator (P. G. and K. S. K.).

### Inclusion and exclusion criteria

Studies were included based on the following criteria: (i) baseline data from observational studies (i.e., cross‐sectional, longitudinal and case–control); (ii) adults irrespective of health status; (iii) adults aged ≥60 years; (iv) clear diagnostic criteria for sarcopenia (i.e., EWGSOP 1 and 2, AWGS, FNIH, CHS); (v) clear criteria for polypharmacy (i.e., ≥5 medications); and (vi) studies had to include both adults with and without sarcopenia. Published articles were excluded if they (i) were reviews, letters, in vivo or in vitro experiments, commentaries or posters; (ii) were not published as a full text and in English; it appears to have little impact on the effect estimates and conclusions of systematic reviews^27^; and (iii) included participants were below 60 years old.

### Data extraction and risk of bias

Two authors (K. P. and K. K. T.) extracted data independently, which included the name of first author, date of publication, country of origin, participant age, study design, population studied, number of participants, health status, prevalence of polypharmacy, number of medications, and definition of polypharmacy and sarcopenia. Disagreements between authors were resolved by two independent reviewers (P. G. and K. S. K.). The quality of the included studies was evaluated using the Methodological index for non‐randomized studies (MINORS) tool^28^ and performed by three independent reviewers (K. P., K. K. T. and K. S. K.). MINORS is a comprehensive tool used to assess bias in nonrandomized controlled trials based on the following items: a clearly stated aim; inclusion of consecutive patients; prospective data collection; endpoints appropriate to study aim; unbiased assessment of study endpoint; follow‐up period appropriate to study aim; <5% lost to follow‐up; prospective calculation of study size; adequate control group; contemporary groups; baseline equivalence of groups; and adequate statistical analyses. According to the scoring system, MINORS' domains are scored as 0 if they are not reported, 1 when they have been reported but with inadequate details, and 2 when they have been reported while providing adequate information. The global ideal score is 16 for noncomparative studies, and a score below 8 was deemed as a high risk of bias and of some concerns, respectively.

### Statistical analysis

Quantitative data were treated as continuous measurements, and changes in outcomes from sarcopenic and non‐sarcopenic individuals were compared between groups to calculate mean differences (MDs) in number of medications and the odds ratio (OR) regarding the prevalence of polypharmacy. When studies provided interquartile ranges (IQR), the formula ‘standard deviation (*SD*) = width of IQR/1.35’ was used to approximately calculate the missing *SD*s.^29^ Statistical significance was assessed using the random‐effects model and inverse‐variance method.

Statistical heterogeneity of outcome measurements between different studies was assessed using the overlap of their confidence interval (95% CI) and expressed as measurements of Cochran's Q (Chi‐square test) and *I* ^2^. The classification of data as having low heterogeneity were based on *I* ^2^ from 30% to 49%, moderate heterogeneity from 50% to 74% and high heterogeneity from 75% and above.^30^ In case of high heterogeneity, a random‐effects meta‐regression was conducted to explore potential sources of variability that could affect estimate rates among studies.^31^ Subgroup analyses based on age, population studied, similar health status, definition of polypharmacy, definition of sarcopenia and geographical location were performed. Moreover, sensitivity analyses were performed to evaluate the robustness of reported statistical results by discounting the effect of existing comorbidities and risk of malnutrition that would differ between sarcopenic and non‐sarcopenic groups on outcome measurements and according to risk of bias of the included cohort studies. Further sensitivity analysis was intended to improve the accuracy of our findings by excluding studies conducted in populations with additional comorbidities in the sarcopenic compared with the group without sarcopenia that could interfere with the number of medications and/or prevalence of polypharmacy. The meta‐analysis was synthesized using Review Manager (RevMan 5.4.1) software. A *P* value of <0.05 was considered statistically significant.

For the evaluation of unexplained variance among studies with substantial heterogeneity, meta‐regressions were performed on the number of medications and prevalence of polypharmacy using a random‐effects model. Six and seven covariates were included in the meta‐regression related to the number of medications and prevalence of polypharmacy, respectively. Particularly, age, population of study, geographical area, risk of bias, muscle mass assessment tool, definition of sarcopenia and definition of polypharmacy were all used as individual covariates using STATA/MP 13.0.

## Results

### Literature search

**Figure 1:**
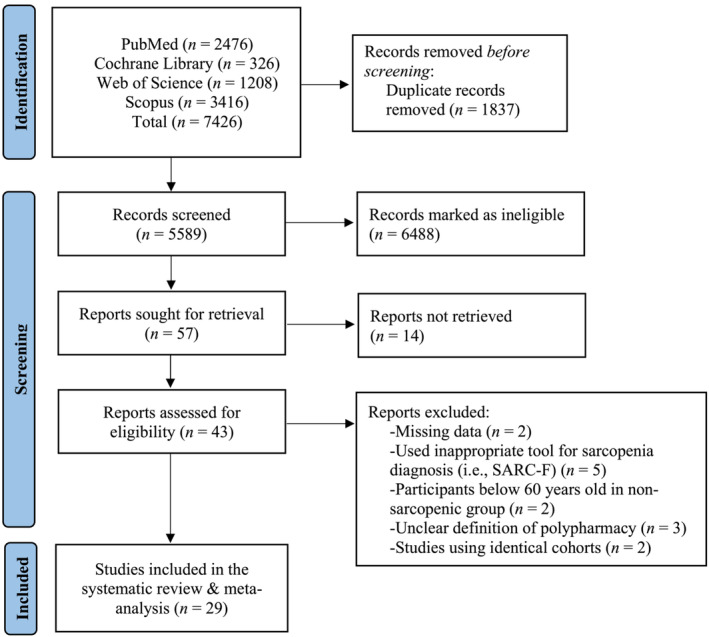
Flowchart of the employed literature search.

The initial literature search provided 7426 publications. Following the exclusion of duplicates and abstracts, 43 full texts were identified as eligible for inclusion in the systematic review and meta‐analysis. Of these 43 studies, two studies were dismissed due to missing data^32^, ^33^ and five studies because of usage of a screening tool for the diagnosis of sarcopenia.^34^, ^35^, ^36^, ^37^, ^38^ Additionally, three studies were excluded due to lack of polypharmacy definition^39^, ^40^, ^41^ and another two^42^, ^43^ due to being part of other studies, albeit one measured the number of medications^44^ while the other polypharmacy prevalence.^45^ In total, 29 studies were included in the systematic review and meta‐analysis exploring the prevalence of polypharmacy and number of medications in people with sarcopenia compared with those without sarcopenia (*Figure* *1*).

### Descriptive results

Seventeen studies assessed the prevalence of polypharmacy^25^, ^45^, ^46^, ^47^, ^48^, ^49^, ^50^, ^51^, ^52^, ^53^, ^54^, ^55^, ^56^, ^57^, ^58^, ^59^, ^60^ and 13 studies assessed the number of medications received.^44^, ^46^, ^61^, ^62^, ^63^, ^64^, ^65^, ^66^, ^67^, ^68^, ^69^, ^70^, ^71^ The study by Suzan et al. was included in the analysis of both groups (prevalence of polypharmacy and number of medications).^46^ Detailed characteristics of the included studies are outlined in *Tables* *1* and *2*.

**Table 1:** Study and participant characteristics of the included studies measuring prevalence of polypharmacy.

				Sarcopenic		Non‐sarcopenic					
Study, year	Country	Study design	Total *n*	*n* (M/F)	Age	*n* (M/F)	Age	Prevalence of Polypharmacy	Polypharmacy Definition	Sarcopenia Definition	Population
Okayama *et al*. 2022	Japan	Cross‐sectional	61	24 (0/24)	81.5 (8.7)	37 (0/37)	75.1 (6.7)	Sarcopenic: 18% Non‐sarcopenic: 16%	≥4 medications	AWGS	Community dwelling
Matsumoto *et al*. 2022	Japan	Retrospective cohort	361	196 (−/−)	78.3 (7.7) All	165 (−/−)	78.3 (7.7) All	Sarcopenic: 58.5% Non‐sarcopenic: 41.5%	≥5 medications	AWGS	Inpatients
Suzan *et al*. 2022	Turkey	Cross‐sectional	258	77 (23/54)	75.7 (8.0)	181 (52/129)	78.4 (7.6)	Sarcopenic: 77% Non‐sarcopenic: 61%	≥5 medications	EWGSOP 2	Community dwelling
Hsu *et al*. 2021	Taiwan	Cross‐sectional	102	47 (40/7)	85.1 (6.2)	55 (38/17)	78.3 (8.5)	Sarcopenic: 36.2% Non‐sarcopenic: 30.9%	≥5 medications	AWGS	Community dwelling
Jang *et al*. 2020	Korea	Longitudinal	1281	370	81.2 (6.6)	911	74.1 (5.7)	Sarcopenic: 29.7% Non‐sarcopenic: 19.4%	≥5 medications	EWGSOP 1 and 2	Community dwelling
Dodds *et al*. 2020	United Kingdom	Cross‐sectional^c^	1686	328 (147/181)	69–70	1358 (677/681)	69–70	Sarcopenic: 40.3% Non‐sarcopenic: 16.2%	≥5 medications	EWGSOP 2^a^	Community dwelling
Sazlina *et al*. 2020	Malaysia	Cross‐sectional^c^	506	144 (69/75)	Multiples ages; most below 80 years	363 (133/229)	Multiple Ages; most below 80 years	Sarcopenic: 25% Non‐sarcopenic: 75%	≥5 medications	AWGS	Community dwelling
Su *et al*. 2019	Japan	Cross‐sectional	310	25 (9/16)	77.8 (5.5)	285 (143/142)	76.0 (5.8)^d^	Sarcopenic: 29.1% Non‐sarcopenic: 52%	≥5 medications	EWGSOP 2	Community dwelling
Agosta *et al*. 2019	Italy	Cross‐sectional	655	227 (115/112)	81–85	428 (200/228)	79–81	Sarcopenic: 59.9% Non‐sarcopenic: 53%	≥5 medications	EWGSOP 1	Inpatients
Kimura *et al*. 2018	Japan	Cross‐sectional	205	30 (8/22)	79.4 (5.0)	175 (67/108)	76.9 (5.1)	Sarcopenic: 63.3% Non‐sarcopenic: 33.7%	≥5 medications	AWGS	Outpatients
Jang *et al*. 2018	Korea	Longitudinal	1343	215 (82/133)	80.8 (6.3)	1128 (520/608)	75.1 (6.1)	Sarcopenic: 34.4% Non‐sarcopenic: 20.1%	≥5 medications	AWGS	Community dwelling
Hao *et al*. 2018	China	Cross‐sectional	407	127 (77/50)	82.0 (8.0)	280 (215/65)	81.0 (8.8)	Sarcopenic: 45% Non‐sarcopenic: 42%	≥5 medications	AWGS	Inpatients
Pérez‐Zepeda *et al*. 2017	Australia	Longitudinal	172	69 (−/−)	85.5 (6.8)	103 (−/−)	85.0 (6.2)	Sarcopenic: 85.5% Non‐sarcopenic: 85.4%	≥6 medications	EWGSOP 1	Inpatients
Yang *et al*. 2017	China	Longitudinal	288	49 (38/11)	83.7 (5.9)	239 (187/52)	80.5 (6.6)	Sarcopenic: 34.7% Non‐sarcopenic: 47.3%	≥5 medications	AWGS	Inpatients
König *et al*. 2017	Germany	Cross‐sectional	1502	127 (78/49)	60–84^e^	1375 (663/712)	60–84^e^	Sarcopenic: 16.3% Non‐sarcopenic: 6.9%	≥5 medications	FNIH Sarcopenia Project^b^	Community dwelling
Yalcin *et al*. 2016	Turkey	Cross‐sectional	141	41 (17/24)	82.6 (7.3)	100 (62/38)	78.0 (7.4)	Sarcopenic: 61% Non‐sarcopenic: 65%	≥5 medications	EWGSOP 1	Nursing home
Hirani *et al*. 2015	Australia	Longitudinal	1496	57 (57/0)	83.5 (6.1)	1439 (1439/0)	76.4 (5.2)	Sarcopenic: 52.6% Non‐sarcopenic: 36.3%	≥5 medications	FNIH Sarcopenic Project	Community dwelling

*Note*: Data are expressed as mean (standard deviation).

Abbreviations: AWGS, Asian Working Group for Sarcopenia; CC, calf circumference; EWGSOP, European Working Group on Sarcopenia in Older People; FNIH, Foundation for the National Institutes of Health Biomarkers Consortium; SARC‐F, strength, assistance with walking, rising from a chair, climbing stairs, and falls.

^a^ Assessed low handgrip strength and chair‐rising test and not muscle mass, indicating probable sarcopenia.

^b^ Assessed low ALM/BMI as part of sarcopenia definition.

^c^ Cross‐sectional data from longitudinal study.

^d^ Mean age of the whole cohort.

^e^ Mean age of the whole cohort (68.7 ± 3.7).

**Table 2:** Study and participant characteristics of the included studies assessing number of medications.

			Total	Sarcopenic		Non‐sarcopenic				
Study, year	Country	Study design	*n*	*n* (M/F)	Age	*n* (M/F)	Age	No of medications	Sarcopenia definition	Population
Formiga *et al*. 2022	Austria, Germany, Israel, Italy, The Netherlands, Poland and Spain	Cross‐sectional	315	41 (23/18)	83.0 (7.0)	274 (146/128)	79.0 (6.0)	Sarcopenic: 9 (4) Non‐sarcopenic: 8 (4.3)	EWGSOP 2	Community dwelling with type 2 diabetes
Remelli *et al*. 2022	Italy	Cross‐sectional	610	139 (89/50)	82.4 (6.8)	471 (208/263)	80.2 (6.5)	Sarcopenic: 6.1 (2.7) Non‐sarcopenic: 6.1 (2.9)	EWGSOP 2	Inpatients
Suzan *et al*. 2022	Turkey	Cross‐sectional	258	77 (23/54)	75.7 (8.0)	181 (52/129)	78.4 (7.6)	Sarcopenic: 77% Non‐sarcopenic: 61%	EWGSOP 2	Community dwelling
Cebria *et al*. 2020	Spain	Cross‐sectional	114	28 (6/22)	>65^≠^	86 (34/52)	>65^c^	Sarcopenic: 8.82 (3.94) Non‐sarcopenic: 0.17 (0.47)	EWGSOP 2	Institutionalized
Curcio *et al*. 2019	Italy	Cross‐sectional	420	55 (−/−)	80.2 (7.3)	365 (79/286)	74.7 (8.2)	Sarcopenic: 4 (1) Non‐sarcopenic: 2 (1)	EWGSOP 1	Inpatients
Pourhassan *et al*. 2018	Germany	Longitudinal	198	50 (20/30)	82.5 (5.7)	148 (39/109)	82.9 (6.0)	Sarcopenic: 8 [5–9]^a^ Non‐sarcopenic: 9 [6–11]^a^	EWGSOP 1	Inpatients
Takahashi *et al*. 2018	Japan	Cross‐sectional	279	86 (23/63)	81.2 (7.4)	193 (83/110)	73.9 (6.4)	Sarcopenic: 6.0 [3.0–7.0]^a^ Non‐sarcopenic: 3.0 [1.0–5.0]^a^	AWGS	Outpatients
Öztürk *et al*. 2018	Turkey	Cross‐sectional	230	61 (40/21)	73.3 (7.2)	169 (92/77)	72.7 (5.9)	Sarcopenic: 3.4 (3.32) Non‐sarcopenic: 3.7 (2.81)	EWGSOP 1	Outpatients
Yalcin *et al*. 2017	Turkey	Cross‐sectional	241	93 (52/41)	83.3 (5.7)	148 (65/83)	79.9 (6.6)	Sarcopenic: 7 [0–13]^a^ Non‐sarcopenic: 6 [0–11]^a^	EWGSOP 1	Nursing home
Beaudart *et al*. 2015	Belgium	Longitudinal	534	73 (25/48)	77.1 (7.0)	461 (187/274)	72.9 (5.8)	Sarcopenic: 6.79 (3.14) Non‐sarcopenic: 5.66 (3.50)	EWGSOP 1	Community dwelling
Gao *et al*. 2015	China	Cross‐sectional	612	60 (7/53)	76.8 (6.7)	552 (247/305)	71.3 (6.4)	Sarcopenic: 2.8 (3.0) Non‐sarcopenic: 2.0 (2.1)	AWGS	Community dwelling
Halil *et al*. 2014	Turkey	Cross‐sectional	711	483 (257/226)	78.5 (7.4)	228 (100/128)	76.3 (7.7)	Sarcopenic: 4.8 (3.0) Non‐sarcopenic: 3.9 (2.3)	Cardiovascular Health Study^b^	Nursing home
Landi *et al*. 2012	Italy	Longitudinal	260	66 (45/21)	86.7 (5.4)	194 (132/62)	84.7 (4.3)	Sarcopenic: 3.1 (1.8) Non‐sarcopenic: 3.0 (2.0)	EWGSOP 1	Community dwelling

*Note*: Data are expressed as mean (standard deviation).

Abbreviations: AWGS, Asian Working Group for Sarcopenia; EWGSOP, European Working Group on Sarcopenia in Older People.

^a^ Median [interquartile range].

^b^ Sarcopenia was defined as low handgrip strength or low calf circumference.

^c^ Mean age of the whole cohort (82.03 ± 8.25).

Five studies were conducted in Japan,^25^, ^47^, ^50^, ^57^, ^69^ five in Turkey,^46^, ^55^, ^66^, ^68^, ^70^ four in Italy,^44^, ^45^, ^63^, ^67^ three in China,^52^, ^53^, ^65^ two in Germany,^54^, ^71^ two in Australia,^56^, ^60^ two in South Korea,^48^, ^51^ one in Spain,^62^ one in United Kingdom,^49^ one in Belgium,^61^ one in Taiwan,^59^ one in Malaysia^58^ and one in multiple European countries.^64^

Eighteen studies were cross‐sectional^44^, ^45^, ^46^, ^47^, ^50^, ^52^, ^54^, ^55^, ^57^, ^59^, ^62^, ^63^, ^64^, ^65^, ^66^, ^68^, ^69^, ^70^ and six longitudinal,^48^, ^51^, ^53^, ^56^, ^61^, ^67^ four studies utilized data from longitudinal studies^49^, ^58^, ^60^, ^71^ and one was a retrospective cohort study.^25^ Cross‐sectional information was collected from all observational studies.

Eighteen studies included subjects with sarcopenia with mean age 80 years and above,^44^, ^45^, ^47^, ^48^, ^51^, ^52^, ^53^, ^55^, ^56^, ^59^, ^60^, ^62^, ^63^, ^64^, ^67^, ^69^, ^70^, ^71^ while 11 studies included subjects with sarcopenia with mean age below 80 years.^25^, ^46^, ^49^, ^50^, ^54^, ^57^, ^58^, ^61^, ^65^, ^66^, ^68^

Subjects with sarcopenia had a greater prevalence of malnutrition in eight studies as opposed to subjects without sarcopenia^46^, ^47^, ^48^, ^53^, ^55^, ^61^, ^69^, ^71^; in three studies, they had greater prevalence of general comorbidities^49^, ^56^, ^62^; in two studies, they had a greater prevalence of depression,^48^, ^67^ a higher prevalence of dementia^45^, ^46^ and type 2 diabetes^46^, ^50^; and in one study, they had higher prevalence of osteoporosis^69^ and respiratory disease.^52^ Nevertheless, nine studies displayed similar rates of general comorbidities between subjects with sarcopenia compared with those without sarcopenia,^44^, ^55^, ^57^, ^59^, ^60^, ^63^, ^65^, ^66^, ^71^ while in four studies, subjects without sarcopenia had a higher prevalence of hypertension^52^, ^53^, ^58^, ^64^; in one study, they had a higher prevalence of type 2 diabetes^58^; and in one study, they had a higher prevalence of chronic kidney disease^52^ compared with subjects with sarcopenia.

Finally, in three studies, there was no report of general comorbidities^25^, ^54^, ^68^ while in two studies, participants with comorbidities were excluded.^47^, ^58^ In 13 studies, subjects were community dwelling^47^, ^48^, ^49^, ^50^, ^51^, ^54^, ^56^, ^58^, ^59^, ^61^, ^64^, ^65^, ^67^; in nine studies, subjects were inpatients^25^, ^44^, ^45^, ^52^, ^53^, ^60^, ^62^, ^63^, ^71^; in four studies, subjects were outpatients^46^, ^57^, ^68^, ^69^; and in three studies, were nursing home residents.^55^, ^66^, ^70^

### Definition of sarcopenia and polypharmacy

To define sarcopenia, nine studies used the EWGSOP 1 criteria,^45^, ^55^, ^60^, ^61^, ^63^, ^67^, ^68^, ^70^, ^71^ and seven studies used the EWGSOP 2 criteria^44^, ^46^, ^48^, ^49^, ^50^, ^62^, ^64^ of which one used low handgrip strength and chair‐rising test performance as a surrogate for probable sarcopenia.^49^ Furthermore, 10 studies used the AWGS criteria,^25^, ^47^, ^51^, ^52^, ^53^, ^57^, ^58^, ^59^, ^65^, ^69^ 2 studies used the FNIH Sarcopenia Project^54^, ^56^ of which one used only appendicular lean mass (ALM) to body mass index (BMI) cut‐off values,^54^ and 1 study used the Cardiovascular Health Study criteria.^66^ Details regarding the diagnostic criteria for sarcopenia in each study are described in *Table* *3*. Polypharmacy was defined as the use of five or more daily medications in 15 studies,^25^, ^45^, ^46^, ^48^, ^49^, ^50^, ^51^, ^52^, ^53^, ^54^, ^55^, ^56^, ^57^, ^58^, ^59^ six or more in 1 study^60^ and four or more in 1 study.^47^

**Table 3:** Diagnostic criteria of sarcopenia in the included studies.

	Criteria	Studies
EWGSOP 1	Grip strength: men, <30 kg; women, <20 kg ASM (BIA): men, <8.87 kg/m^2^; women, <6.42 kg/m^2^ Gait speed: ≤0.8 m/s	^45^, ^55^, ^63^, ^68^, ^70^
	Grip strength: men, <30 kg; women, <20 kg ASM (BIA): men, <8.87 kg/m^2^; women, <6.42 kg/m^2^ SPPB: ≤8 point score	^71^
	Grip strength: Lowest quintile adjusted for BMI & sex ASM (BIA): men, <10.76 kg/m^2^; women, <6.76 kg/m^2^	^60^
	Grip strength: men, <30 kg; women, <20 kg ASM (DXA): men, <7.26 kg/m^2^; women, <5.5 kg/m^2^ SPPB: ≤8 point score	^61^
	Grip strength: men, <30 kg; women, <20 kg Gait speed: ≤0.8 m/s Mid‐arm circumference: men, <21.1 cm; women, <19.2 cm	^67^
EWGSOP 2	Grip strength: men, <27 kg; women, <16 kg ASM (BIA): men, <20 kg; women, <15 kg	^46^
	Grip strength: men, <27 kg; women, <16 kg ASM (BIA): men, <20 kg; women, <15 kg SPPB: ≤8 point score	^64^
	Grip strength: men, <27 kg; women, <16 kg ASM (BIA)/height^2^: men <7.0 kg/m^2^; women, <6.0 kg/m^2^ SPPB: ≤8 point score	^62^
	Grip strength: men, <27 kg; women, <16 kg ASM (BIA): men, <20 kg; women, <15 kg SPPB: ≤8 point score Gait speed: ≤0.8 m/s	^48^
	Grip strength: men, <27 kg; women, <16 kg Chair stand; >15 sec for five rises	^49^
	Grip strength: men, <27 kg; women, <16 kg ASM (BIA)/height^2^: men <7.0 kg/m^2^; women, 5.5 kg/m^2^	^44^, ^50^
AWGS	Grip strength: women, <18 kg ASM (BIA): women, <5.7 kg/m^2^ 5‐m chair stand test: ≥12 s	^47^
	Grip strength: men, <26 kg; women, <18 kg ASM (BIA): men, <7.0 kg/m^2^; women, <5.7 kg/m^2^ Gait speed: ≤0.8 m/s	^57^, ^58^, ^59^
	Grip strength: men, <26 kg; women, <18 kg CC: men, <34 cm; women, <33 cm Gait speed: ≤0.8 m/s	^69^
	ASM (BIA)/BMI: men, <0.789; women, <0.512 Grip strength: men, <26 kg; women, <18 kg Gait speed: ≤0.8 m/s	^51^
	CC: <31 cm Grip strength: men, <26 kg; women, <18 kg Gait speed: ≤0.8 m/s	^52^, ^65^
	Grip strength: men, <26 kg; women, <18 kg ASM (BIA): men, <6.7 kg/m^2^; women, <4.75 kg/m^2^ Gait speed: ≤0.8 m/s	^53^
	Grip strength: men, <26 kg; women, <18 kg ASM (BIA): men, <7.0 kg/m^2^; women, <5.7 kg/m^2^	^25^
FNIH	ASM (DXA)/BMI: men, <0.789; women, <0.512	^54^
	ASM (DXA): men, 19.75 kg Grip strength: men, <26 kg Gait speed: ≤0.8 m/s	^56^
CHS	CC: <31 cm Grip strength: men (BMI ≤ 24 → ≤29 kg; BMI 24.1–28 → ≤30 kg; BMI > 28; ≤32 kg) women (BMI ≤ 23 → ≤17 kg; BMI 23.1–26 → ≤17.3 kg; BMI 26.1–29 → ≤18 kg; BMI > 29; ≤21 kg)	^66^

Abbreviations: ASM, appendicular skeletal muscle; AWGS, Asian Working Group for Sarcopenia; BIA, bioelectrical impedance; BMI, body mass index; CC, calf circumference; CHS, Cardiovascular Health Study; DXA, dual‐energy x‐ray absorptiometry; EWGSOP, European Working Group on Sarcopenia in Older People; FNIH, Foundation for the National Institutes of Health Biomarkers Consortium Sarcopenia Project; SPPB, short physical performance battery.

### Polypharmacy in older subjects with sarcopenia versus without sarcopenia

**Figure 2:**
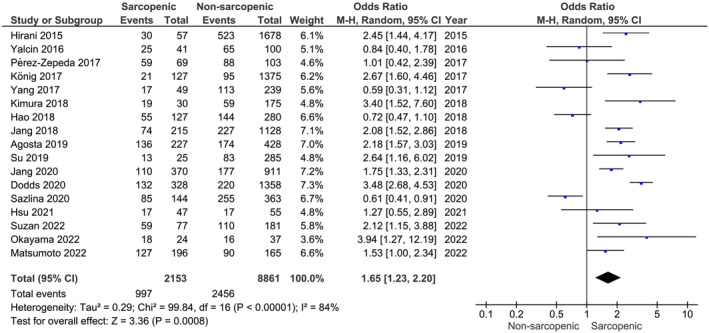
Association of polypharmacy in subjects with sarcopenia versus without sarcopenia.

Our main analysis (*k* = 17, 2153 subjects with sarcopenia and 8861 subjects without sarcopenia) showed that sarcopenia was associated with a higher prevalence of polypharmacy (OR: 1.65, 95% CI [1.23, 2.20], *I* ^2^ = 84%, *P* < 0.01) (*Figure* *2*).

Based on age, we observed a significant risk of having polypharmacy in subjects with sarcopenia below 80 years of age (*k* = 9, OR: 2.02, 95% CI [1.36, 3.01], *I* ^2^ = 86%, *P* < 0.01), although no changes were observed in subjects 80 years and above (*k* = 8, OR: 1.27, 95% CI [0.82, 1.97], *I* ^2^ = 80%, *P* = 0.28) (*Figure* *S1*).

According to similar health status, we did not find a greater risk of polypharmacy in sarcopenic versus non‐sarcopenic groups (*k* = 4, OR: 1.38, 95% CI [0.74, 2.56], *I* ^2^ = 57%, *P* = 0.31) (*Figure* *S2*).

In addition, a significant risk in having polypharmacy was observed in community‐dwelling subjects with sarcopenia (*k* = 9, OR: 2.00, 95% CI [1.35, 2.97], *I* ^2^ = 86%, *P* < 0.01) and outpatients (*k* = 2, OR: 2.51, 95% CI [1.55, 4.07], *I* ^2^ = 0%, *P* < 0.01), but not in hospital inpatients (*k* = 5; OR: 1.11, 95% CI [0.65, 1.89], *I* ^2^ = 83%, *P* = 0.70) (*Figure* *S3*).

In Europe, a significant higher risk of having polypharmacy in subjects with sarcopenia was found (*k* = 5, OR: 2.23, 95% CI [1.52, 3.27], *I* ^2^ = 73%, *P* < 0.01), but not in Asia (*k* = 10, OR: 1.42, 95% CI [0.97, 2.07], *I* ^2^ = 83%, *P* = 0.07) (*Figure* *S4*).

In terms of sarcopenia definition, a significant risk in having polypharmacy was found in older adults with sarcopenia following the EWGSOP criteria (*k* = 7, OR: 1.95, 95% CI [1.38, 2.76], *I* ^2^ = 75%, *P* < 0.01); however, this risk between sarcopenic and non‐sarcopenic groups was not displayed in studies that used the AWGS criteria (*k* = 8, OR: 1.30, 95% CI [0.81, 2.08], *I* ^2^ = 84%, *P* = 0.28) (*Figure* *S5*).

Furthermore, based on muscle mass assessment tool, a significant difference was depicted between groups evaluated via both bioelectrical impedance (BIA) (*k* = 12, OR: 1.54, 95% CI [1.13, 2.11], *I* ^2^ = 78%, *P* < 0.01) and dual‐energy x‐ray absorptiometry (DXA) (*k* = 3, OR: 2.25, 95% CI [1.55, 3.27], *I* ^2^ = 17%, *P* < 0.01) (*Figure* *S6*).

Finally, based on the polypharmacy definition, five or more medications posed a significant risk in having polypharmacy in older adults with sarcopenia versus without sarcopenia (*k* = 15, OR: 1.63, 95% CI [1.20, 2.21], *I* ^2^ = 85%, *P* < 0.01) (*Figure* *S7*). Details of subgroup analysis are found in *Table* *S2*.

Following multiple sensitivity analyses, no changes from the main analysis were detected following the deletion of studies including no report of fully appropriate criteria for sarcopenia definition (OR: 1.49, 95% CI [1.12, 1.98], *I* ^2^ = 79%, *P* < 0.01) (*Figure* *S8*), dementia (OR: 1.58, 95% CI [1.13, 2.21], *I* ^2^ = 86%, *P* < 0.01) (*Figure* *S9*), depression (OR: 1.64, 95% CI [1.18, 2.27], *I* ^2^ = 85%, *P* < 0.01) (*Figure* *S10*), type 2 diabetes (OR: 1.58, 95% CI [1.15, 2.17], *I* ^2^ = 86%, *P* < 0.01) (*Figure* *S11*), respiratory disease (OR: 1.75, 95% CI [1.32, 2.32], *I* ^2^ = 81%, *P* < 0.01) (*Figure* *S12*), prevalence of malnutrition (OR: 1.74, 95% CI [1.21, 2.50], *I* ^2^ = 87%, *P* < 0.01) (*Figure* *S13*), and high risk of bias (OR: 1.61, 95% CI [1.18, 2.21], *I* ^2^ = 85%, *P* < 0.01) (*Figure* *S14*).

### Number of medications in older subjects with sarcopenia versus those without sarcopenia

**Figure 3:**
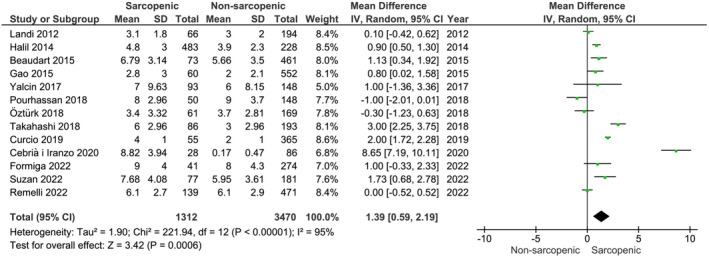
Association of number of medications in subjects with sarcopenia versus without sarcopenia.

Our main analysis (*k* = 13; 1312 subjects with sarcopenia and 3470 subjects without sarcopenia) showed that the number of medications was significantly higher in older subjects with sarcopenia compared with those without sarcopenia (MD: 1.39, 95% CI [0.59, 2.19], *I* ^2^ = 95%, *P* < 0.01) (*Figure* *3*).

Based on subgroup analyses, we observed a significantly higher number of medications in both subjects with sarcopenia and without sarcopenia below 80 years of age (*k* = 5, MD: 0.85, 95% CI [0.34, 1.35], *I* ^2^ = 56%, *P* < 0.01) and 80 years and above (*k* = 8, MD: 1.79, 95% CI [0.49, 3.09], *I* ^2^ = 97%, *P* < 0.01) (*Figure* *S15*).

In subjects with similar health status, no differences regarding the number of medications were observed between individuals with versus without sarcopenia (*k* = 5, MD: 0.60, 95% CI [−0.35, 1.55], *I* ^2^ = 94%, *P* = 0.22) (*Figure* *S16*).

In addition, a significantly greater number of medications was found in community‐dwelling (*k* = 4, MD: 0.66, 95% CI [0.11, 1.21], *I* ^2^ = 48%, *P* = 0.02) and nursing home residents (*k* = 2, MD: 0.90, 95% CI [0.51, 1.30], *I* ^2^ = 0%, *P* < 0.01), but not in inpatients (*k* = 4, MD: 2.31, 95% CI [0.04, 4.57], *I* ^2^ = 98%, *P* = 0.05) and outpatients (*k* = 3, MD: 1.49, 95% CI [−0.51, 3.48], *I* ^2^ = 93%, *P* = 0.14) (*Figure* *S17*).

In Europe, a significant difference in number of medications was detected in subjects with sarcopenia (*k* = 11, MD: 1.30, 95% CI [0.41, 2.19], *I* ^2^ = 95%, *P* < 0.01); however, a significant difference between groups was not established in Asia (*k* = 2, MD: 1.90, 95% CI [−0.25, 4.06], *I* ^2^ = 94%, *P* = 0.08) (*Figure* *S18*).

Moreover, based on muscle mass assessment tool, significant differences were observed between groups via both BIA (*k* = 8, MD: 1.58, 95% CI [0.19, 2.98], *I* ^2^ = 96%, *P* = 0.03) and following measurements of calf circumference (*k* = 3, MD: 1.55, 95% CI [0.26, 2.84], *I* ^2^ = 92%, *P* = 0.02) (*Figure* *S19*).

In terms of sarcopenia definition, a significant difference was found in subjects with sarcopenia versus without sarcopenia following the EWGSOP criteria (*k* = 11, MD: 1.36, 95% CI [0.29, 2.43], *I* ^2^ = 95%, *P* = 0.01); however, no changes were observed when the AWGS criteria were used (*k* = 2, MD: 1.90, 95% CI [−0.25, 4.06], *I* ^2^ = 94%, *P* = 0.08) (*Figure* *S20*). Details of subgroup analysis are found in *Table* *S3*.

Following multiple sensitivity analyses, no changes from the main analysis were detected following the deletion of studies that included subjects with osteoporosis (MD: 1.25, 95% CI [0.43, 2.07], *I* ^2^ = 95%, *P* < 0.01) (*Figure* *S21*), depression (MD: 1.51, 95% CI [0.66, 2.37], *I* ^2^ = 95%, *P* < 0.01) (*Figure* *S22*), dementia and type 2 diabetes (MD: 1.37, 95% CI [0.52, 2.21], *I* ^2^ = 95%, *P* < 0.01) (*Figure* *S23*), sarcopenia definition (MD: 1.45, 95% CI [0.52, 2.38], *I* ^2^ = 95%, *P* < 0.01) (*Figure* *S24*), prevalence of malnutrition (MD: 1.47, 95% CI [0.48, 2.45], *I* ^2^ = 96%, *P* < 0.01) (*Figure* *S25*) and high risk of bias (MD: 1.41, 95% CI [0.59, 2.24], *I* ^2^ = 95%, *P* < 0.01) (*Figure* *S26*).

### Meta‐regression analyses

The increased heterogeneity observed for the prevalence of polypharmacy and number of medications in individuals with vs. without sarcopenia was further investigated via meta‐regression analyses. Differences in study population, age, risk of bias, geographical location, sarcopenia and polypharmacy definition, and muscle mass assessment tool did not explain the potentially high heterogeneity among studies in individuals with and without sarcopenia.

Regarding the prevalence of polypharmacy between individuals with and without sarcopenia, variance among studies was observed due to different populations (i.e., community dwelling, nursing home residents, inpatients and outpatients) (*r* = −0.338, SE = 0.1669, 95% CI [−0.67, −0.01], *z* = −2.03, *P* = 0.04), while no other covariates (i.e., age, risk of bias, geographical location, sarcopenia definition and muscle mass assessment tool) were considered potential sources of variance among included studies. Similarly, in studies measuring the number of medications, variance was explained due to different population groups (*r* = 0.589, SE = 0.2615, 95% CI [0.08, 1.10], *z* = 2.25, *P* = 0.02). Meta‐regression analysis according to participant health status was not feasible, considering the heterogeneity of different comorbidities assessed in multiple studies.

### Risk of bias of included studies

The overall quality of the included studies was considered high (*Table* *S4*), although two studies were considered having a high risk of bias.^45^, ^70^

## Discussion

In this systematic review with meta‐analysis, including 29 studies, we found that older subjects with sarcopenia had an increased prevalence of polypharmacy and a mean higher number of medications compared with those without sarcopenia. The meta‐regression analysis explained only a limited part of the heterogeneity found.

Both polypharmacy and sarcopenia are very common conditions among older adults.^9^ Overall, the findings of the present study suggest that among sarcopenic older adults, the number of medications is, in mean, 1.39 higher and the prevalence of polypharmacy is 65% higher, compared with their counterparts without sarcopenia. Of importance, the association between sarcopenia and polypharmacy seems to be significant among outpatients and community dwellers, but not in hospitalized people and nursing home patients. Moreover, the diagnostic criteria used seem another important factor to consider because the association with sarcopenia was observed when this condition was diagnosed using EWGSOP criteria, but not those proposed by AWGS underlining new potential areas of research. However, it is worth noting that this observation may also be attributed to differences in populations due to variety of ethnicities and geographical areas.

Ageing itself may increase the vulnerability to a variety of adverse effects,^72^ further complicating the importance of polypharmacy in older people having sarcopenia. In this regard, even if the studies included did not report detailed information regarding the categories of medications used, we can argue that certain medications may influence body composition, whereas other drugs may have negative effects on muscles mainly leading to malnutrition (e.g., increasing nausea or diarrhoea).^9^ Traditionally, some medications commonly used in older people, such as glucocorticoids and antidepressants are associated with muscle toxicity, whereas others (e.g., beta blockers or non‐steroidal anti‐inflammatory drugs [NSAIDS]) might cause detrimental metabolic effects, such as mitochondrial dysfunction, diminished blood flow and electrolyte, hormonal or acid–base alterations.^54^ Further, they may also suppress acute exercise‐induced responses via reduction of gene transcription regulators such as mitogen‐activated protein kinases (MAPKs) and nuclear factor‐kappa beta (NF‐κB),^73^ necessary for exercise‐stimulated adaptations of the skeletal muscle.^74^ These chronic alterations may lead to blunted strength and muscle hypertrophic adaptations,^75^ although research in this area is inconclusive.^76^

Generally, polypharmacy can only be considered as an indicator for several adverse clinical outcomes,^6^ even if a direct relationship with these outcomes has not been yet established.^9^ It is therefore imperative to assess whether polypharmacy may play a causative role in the occurrence of sarcopenia since sarcopenia itself is associated with an increased risk of several devastating health outcomes, including disability, higher rate of surgical complications and increased mortality.^77^ At present, we can hypothesize that the association between polypharmacy and sarcopenia might be mediated by other conditions, in particular malnutrition.^26^

### Strengths and limitations

This is the first study assessing quantitatively the relationship of polypharmacy in people living with sarcopenia and those without. We could not reliably assess publication bias because observational studies that report prevalence rates do not indicate positive or negative results, and there is currently no established method to test for publication bias. In addition, publication bias could not have been established also due to the small number of studies per definition of sarcopenia classification (i.e., EWGSOP 1 and 2) and the high heterogeneity among studies. Based on current recommendations, if the heterogeneity is high, at least 10 studies are needed to examine reporting bias using funnel plots.^78^ Furthermore, we found that those with sarcopenia below 80 years old were at an increased risk of polypharmacy versus those without sarcopenia, albeit no significant differences were found among individuals aged 80 years and above. These results raise questions in relation to the confounding factors that could have mediated this relationship between the two ageing groups; however, these covariates were not available to be accounted for in the analyses. Accordingly, a critical limitation is the lack of information on medications compiling polypharmacy in the studies included in our analysis. For example, certain medications may improve an individual's condition that may (in)directly reduce the risk of sarcopenia; hence, attention should be raised regarding appropriate versus inappropriate drug prescription. Finally, it is worth stating the possible inflation pertaining to the number of medications, which could overestimate polypharmacy status, considering the errors that may emerge by inaccurate coding of drug prescription and/or incorrect tabulations conducted electronically.

## Conclusions

This systematic review and meta‐analysis clearly reported that the problem of polypharmacy in people with sarcopenia may be critical, considering its significantly increased prevalence compared with populations without sarcopenia. Polypharmacy is associated with several negative clinical outcomes in older people, including sarcopenia, raising critical questions regarding appropriate versus inappropriate drug prescription. Future research should clarify if polypharmacy is a direct contributor in accelerating the progression of sarcopenia and if inappropriate deprescribing could decelerate its progression.

## Conflict of interest

The authors declare no conflicts of interest.

## Supporting information


**Figure S1.** Supporting information

**Figure S2.** Supporting information

**Figure S3.** Supporting information

**Figure S4.** Supporting information

**Figure S5.** Supporting information

**Figure S6.** Supporting information

**Figure S7.** Supporting information

**Figure S8.** Supporting information

**Figure S9.** Supporting information

**Figure S10.** Supporting information

**Figure S11.** Supporting information

**Figure S12.** Supporting information

**Figure S13.** Supporting information

**Figure S14.** Supporting information

**Figure S15.** Supporting information

**Figure S16.** Supporting information

**Figure S17.** Supporting information

**Figure S18.** Supporting information

**Figure S19.** Supporting information

**Figure S20.** Supporting information

**Figure S21.** Supporting information

**Figure S22.** Supporting information

**Figure S23.** Supporting information

**Figure S24.** Supporting information

**Figure S25.** Supporting information

**Figure S26.** Supporting information

**Table S1.** Supporting information

**Table S2 and Table S3.** Supporting information

**Table S4.** Supporting information
