# Supplementary figures and images for: Sarcopenia is associated with a greater risk of polypharmacy and number of medications: a systematic review and meta‐analysis
Source: J Cachexia Sarcopenia Muscle. 2023 Feb 13;14(2):671–83. doi: 10.1002/jcsm.13190 (PMC10067503; doi:10.1002/jcsm.13190)

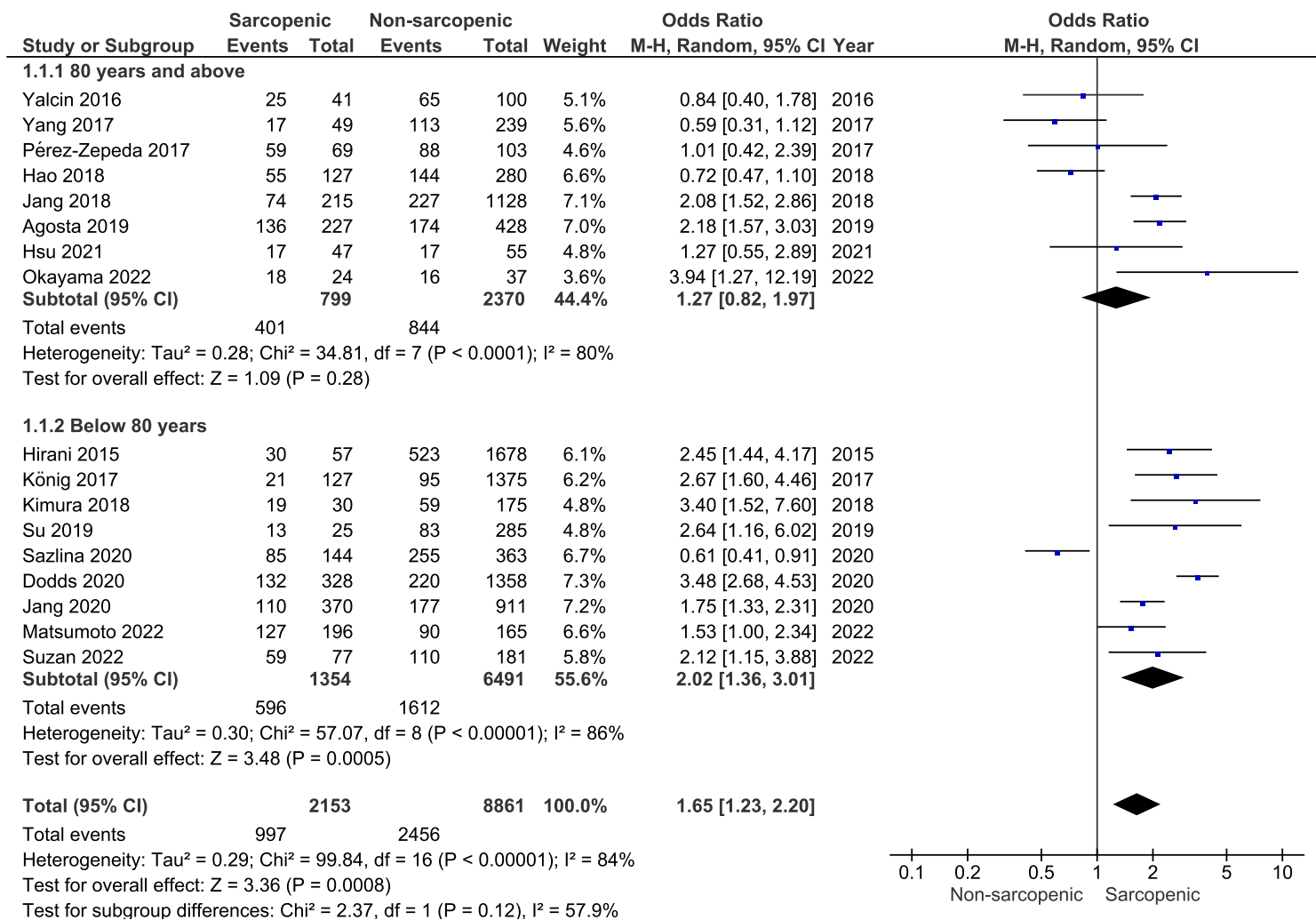

Supplement: Supplementary file 1 — Figure S1. Supporting information [file JCSM-14-671-s021.pdf]

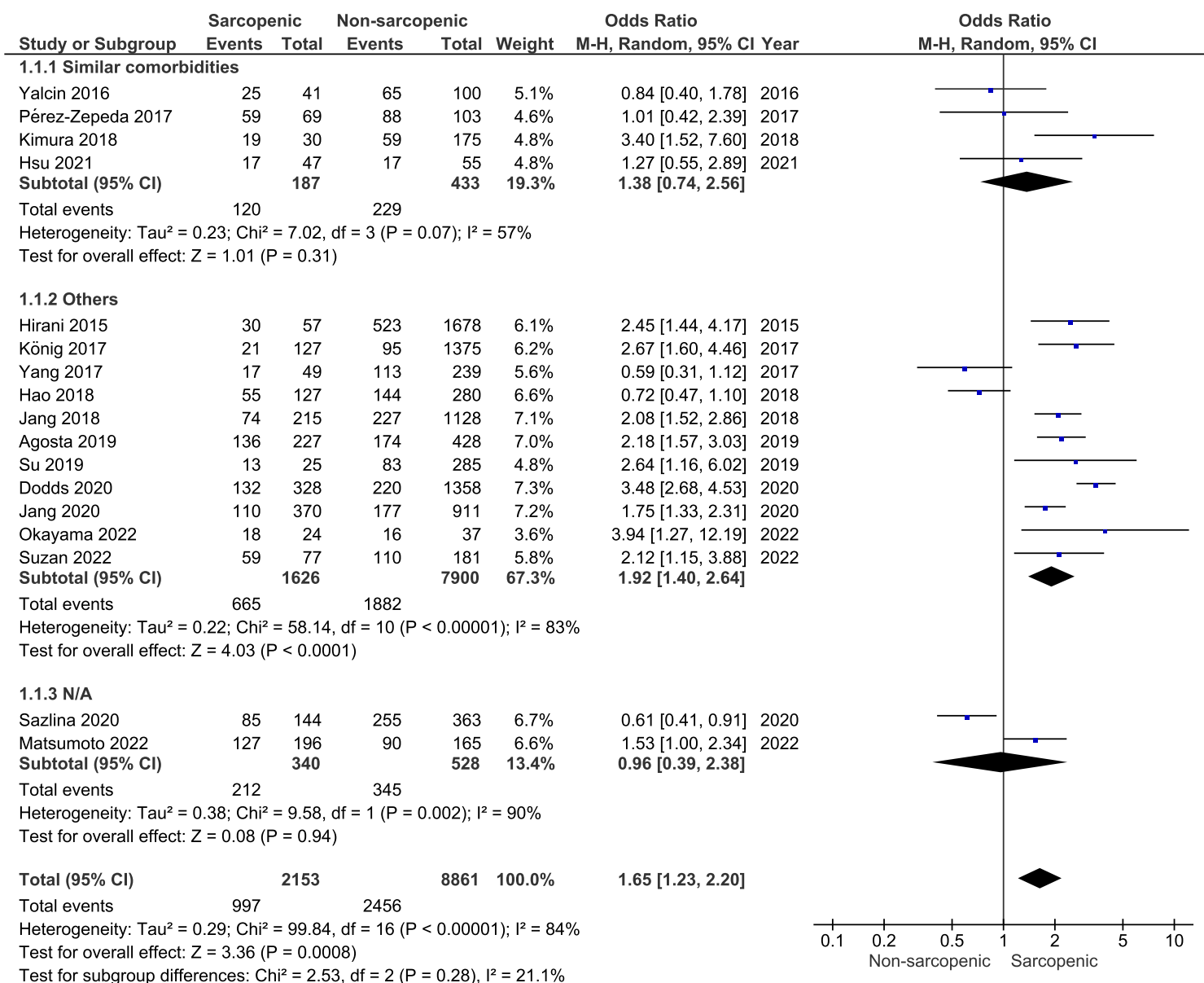

Supplement: Supplementary file 2 — Figure S2. Supporting information [file JCSM-14-671-s028.pdf]

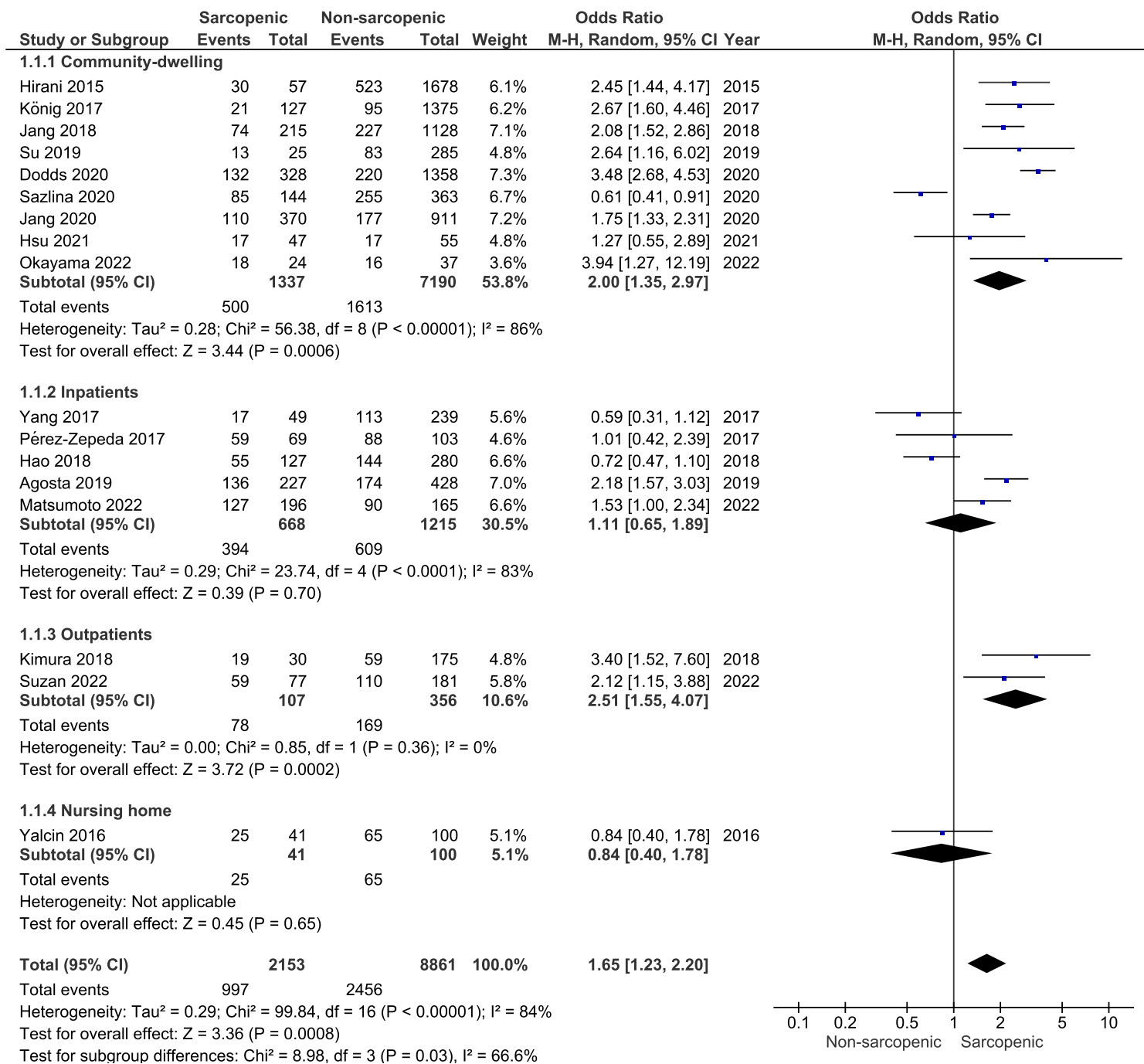

Supplement: Supplementary file 3 — Figure S3. Supporting information [file JCSM-14-671-s013.pdf]

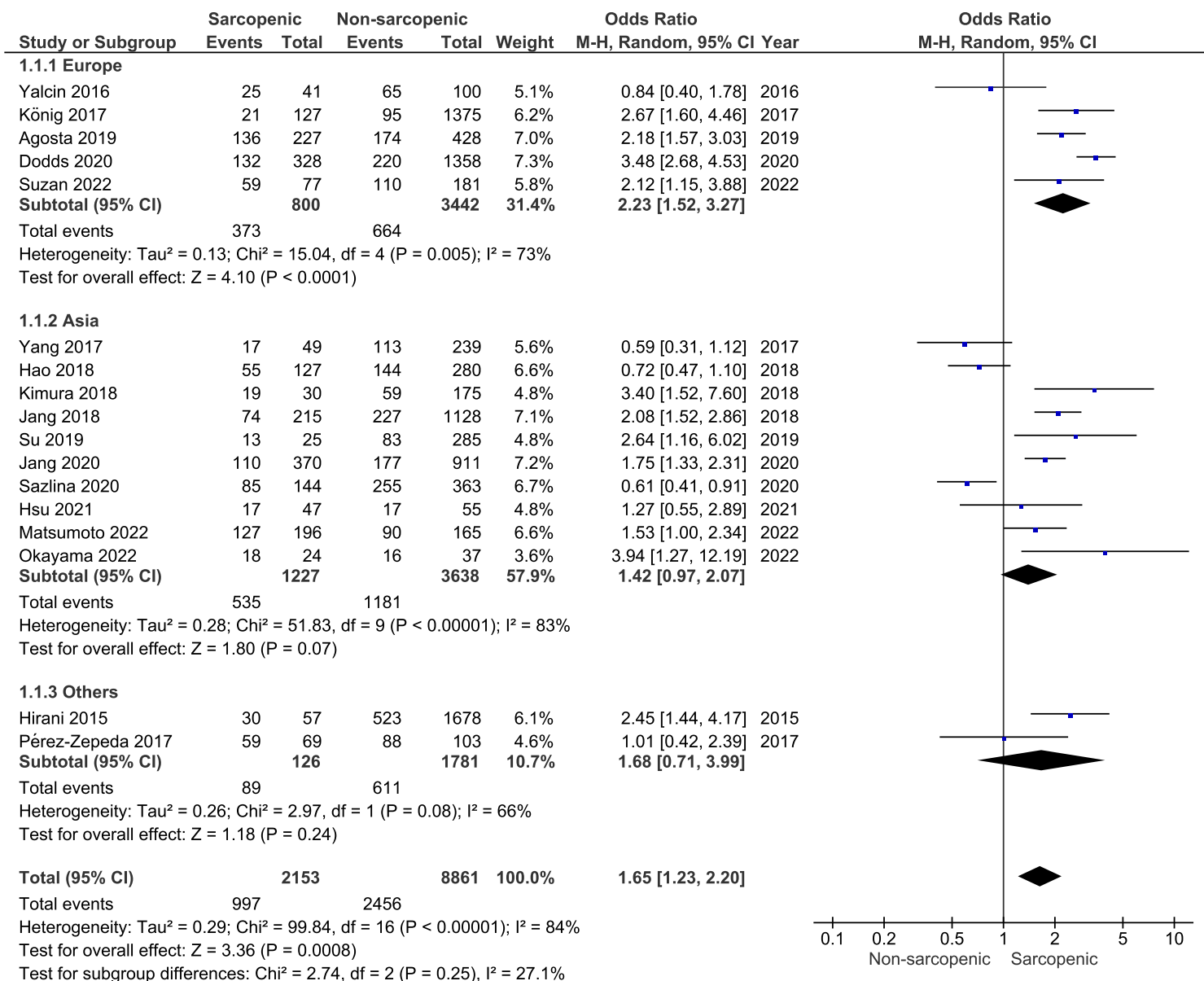

Supplement: Supplementary file 4 — Figure S4. Supporting information [file JCSM-14-671-s003.pdf]

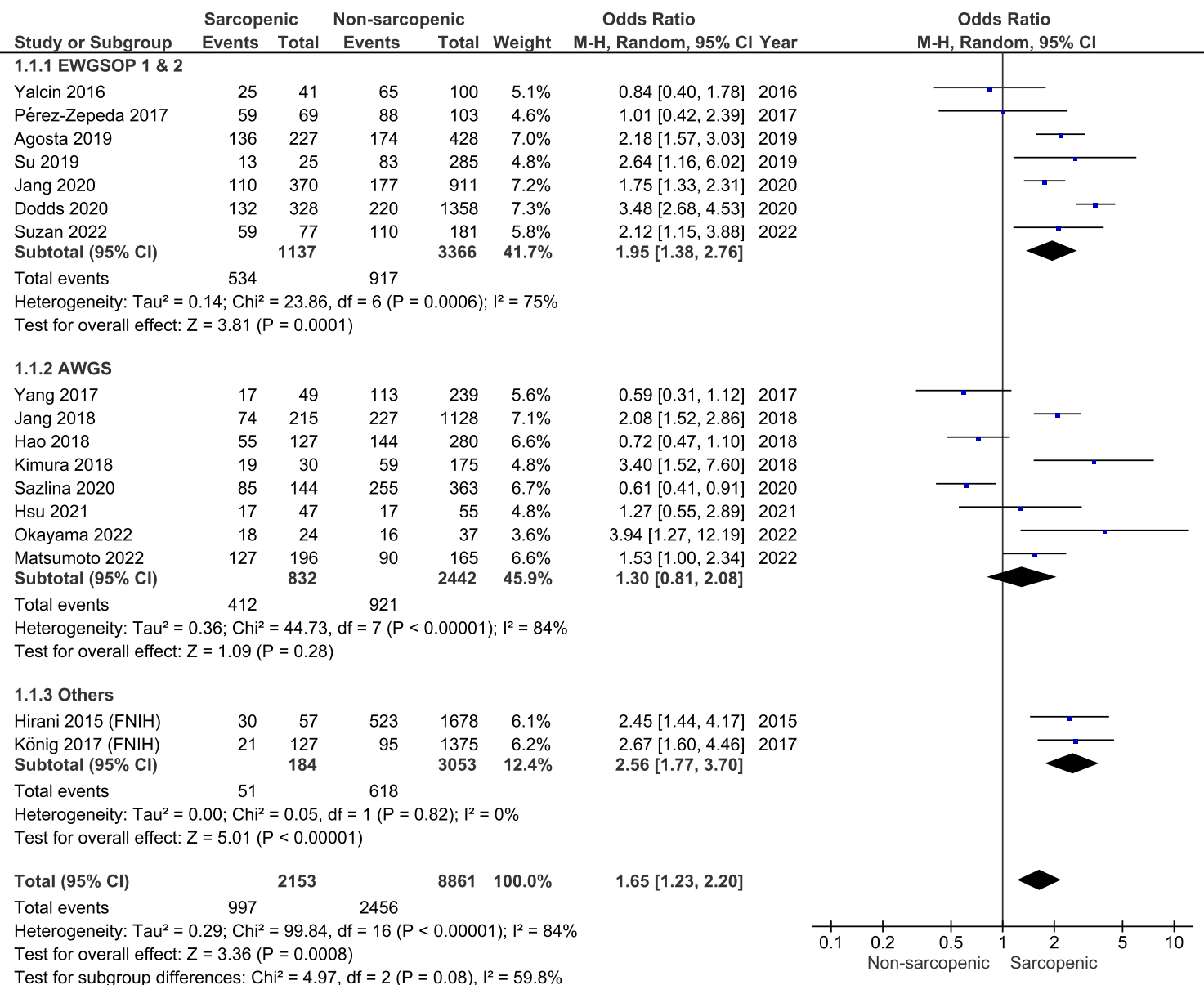

Supplement: Supplementary file 5 — Figure S5. Supporting information [file JCSM-14-671-s026.pdf]

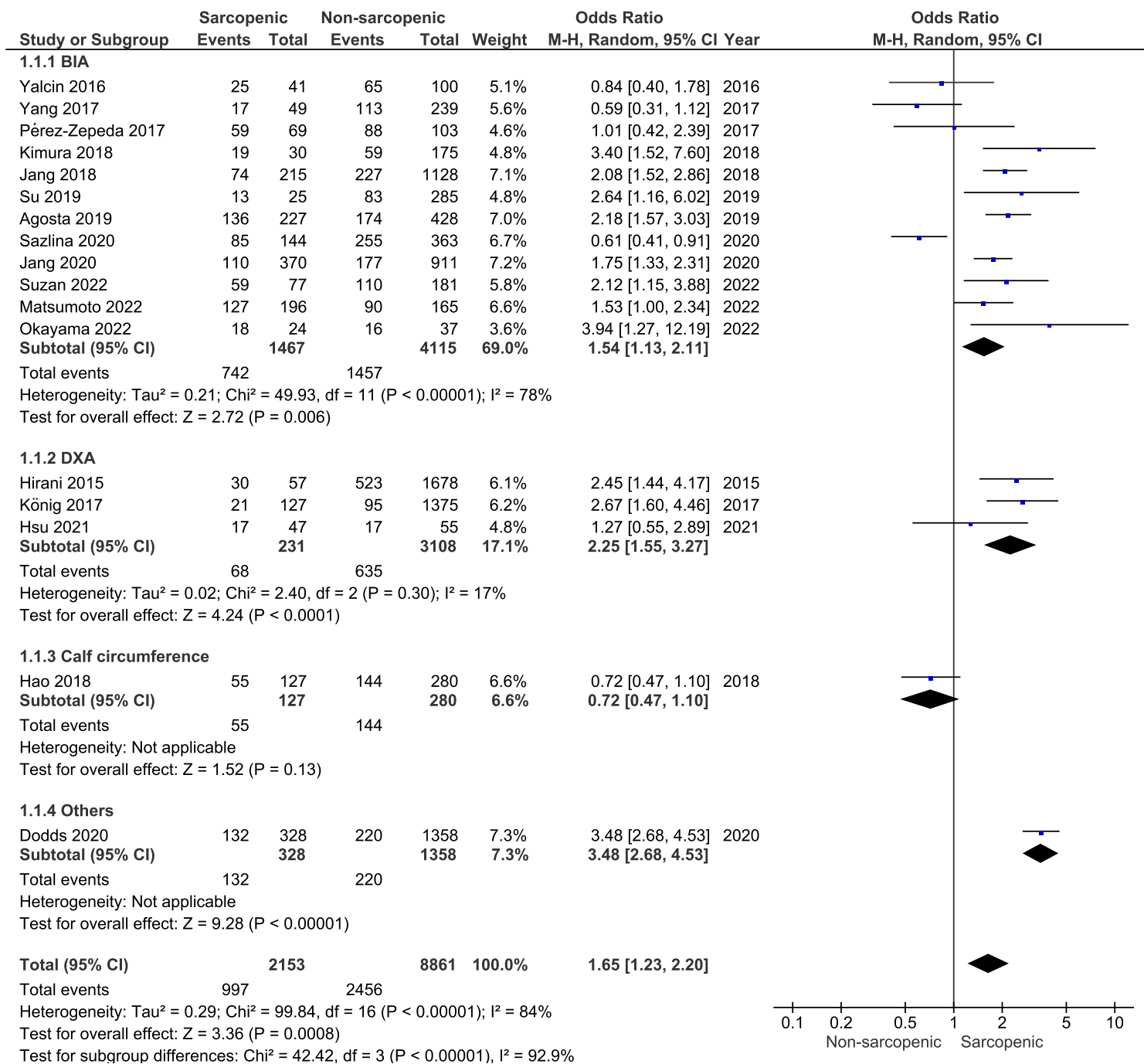

Supplement: Supplementary file 6 — Figure S6. Supporting information [file JCSM-14-671-s015.pdf]

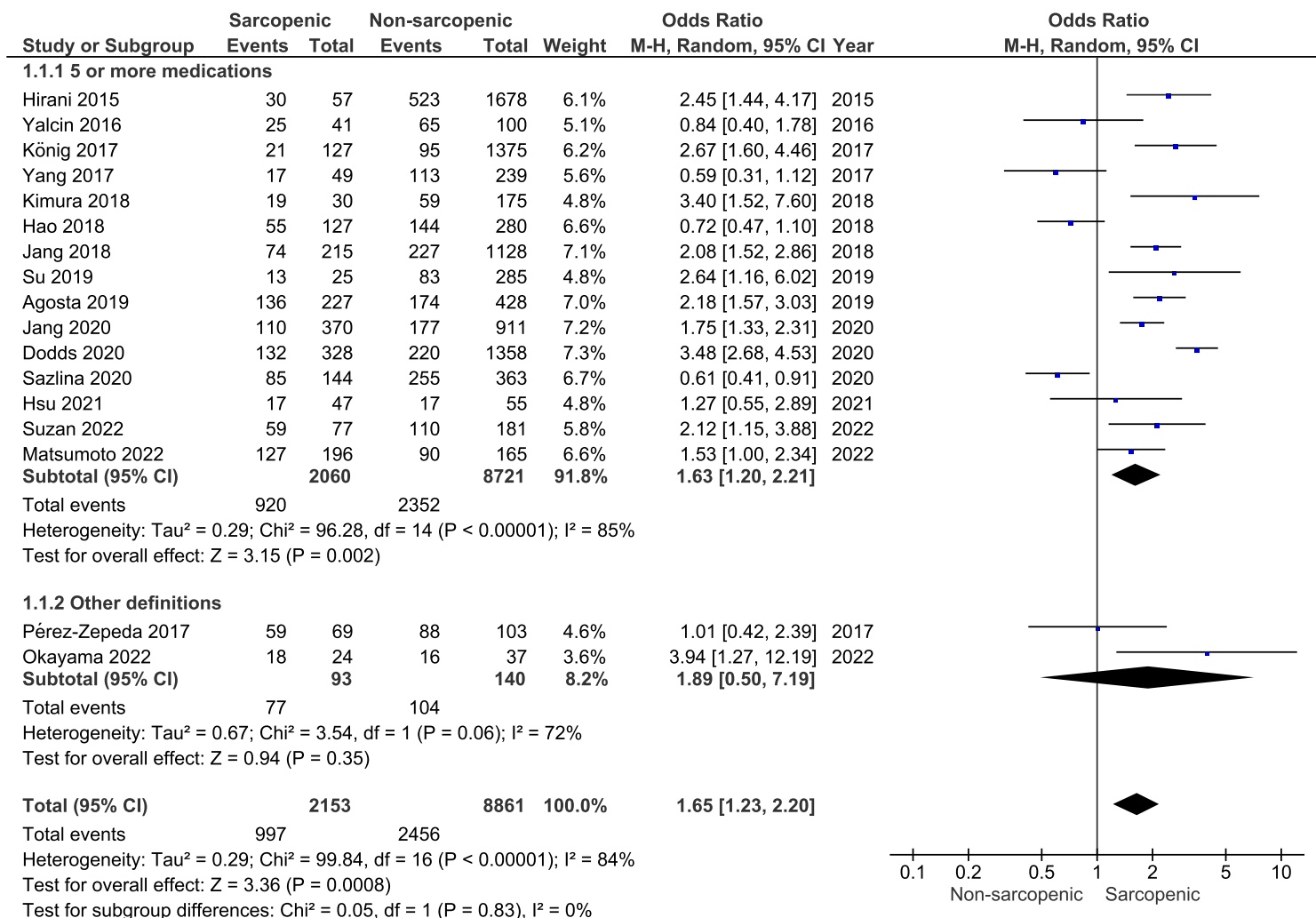

Supplement: Supplementary file 7 — Figure S7. Supporting information [file JCSM-14-671-s010.pdf]

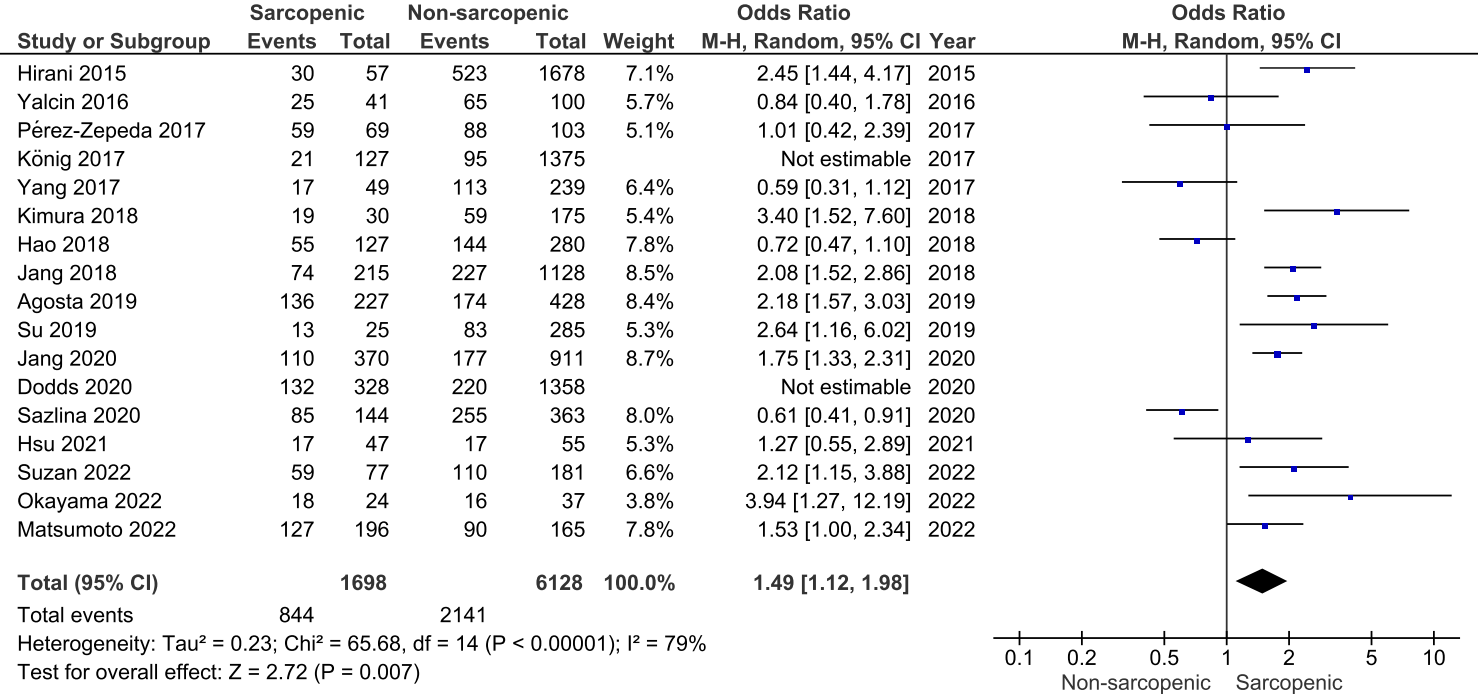

Supplement: Supplementary file 8 — Figure S8. Supporting information [file JCSM-14-671-s025.pdf]

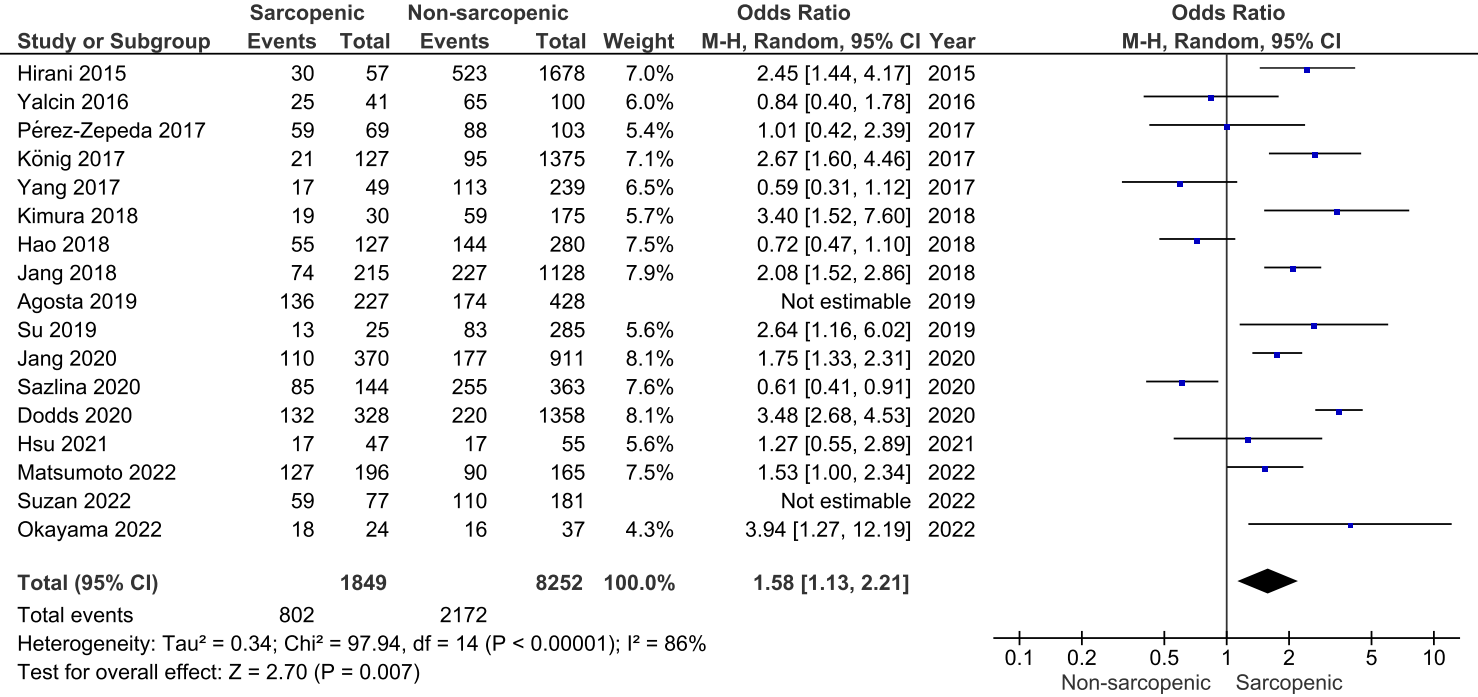

Supplement: Supplementary file 9 — Figure S9. Supporting information [file JCSM-14-671-s001.pdf]

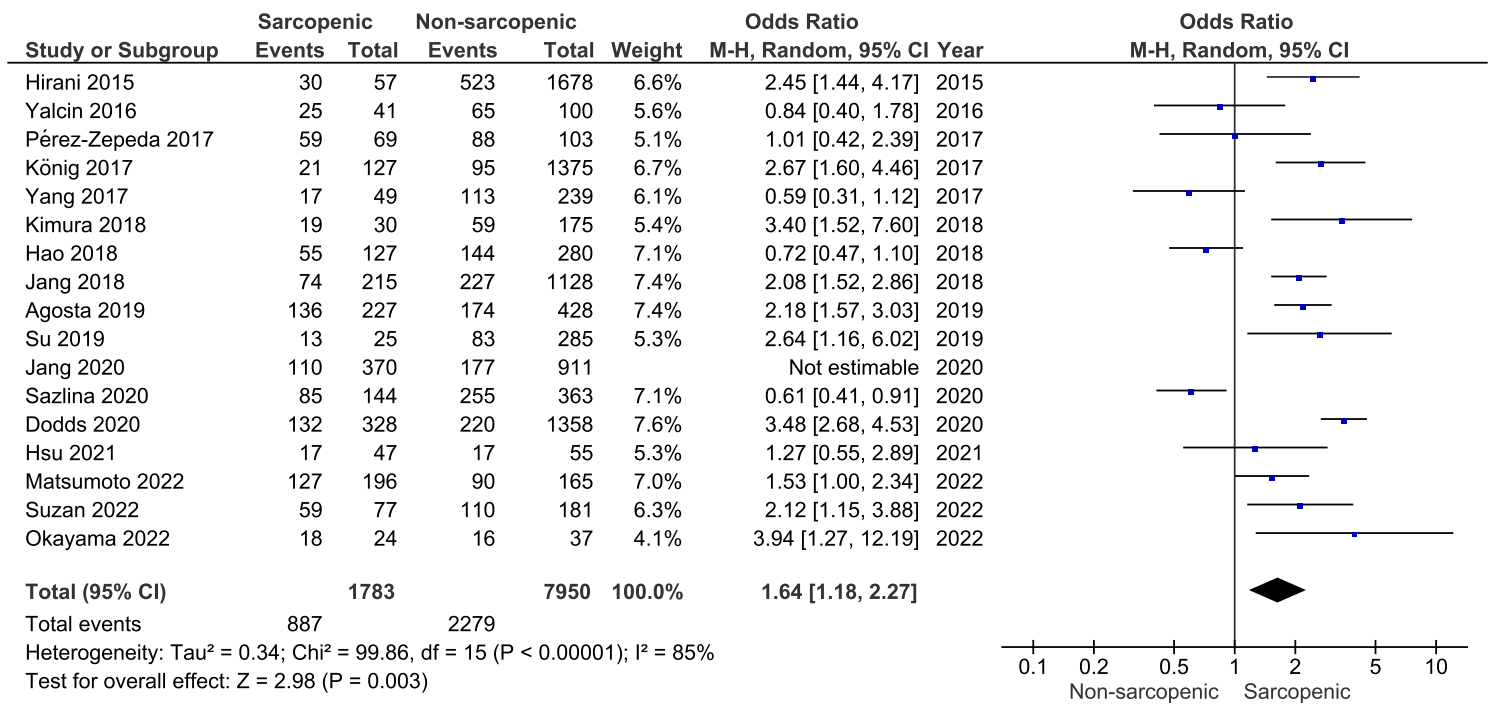

Supplement: Supplementary file 10 — Figure S10. Supporting information [file JCSM-14-671-s012.pdf]

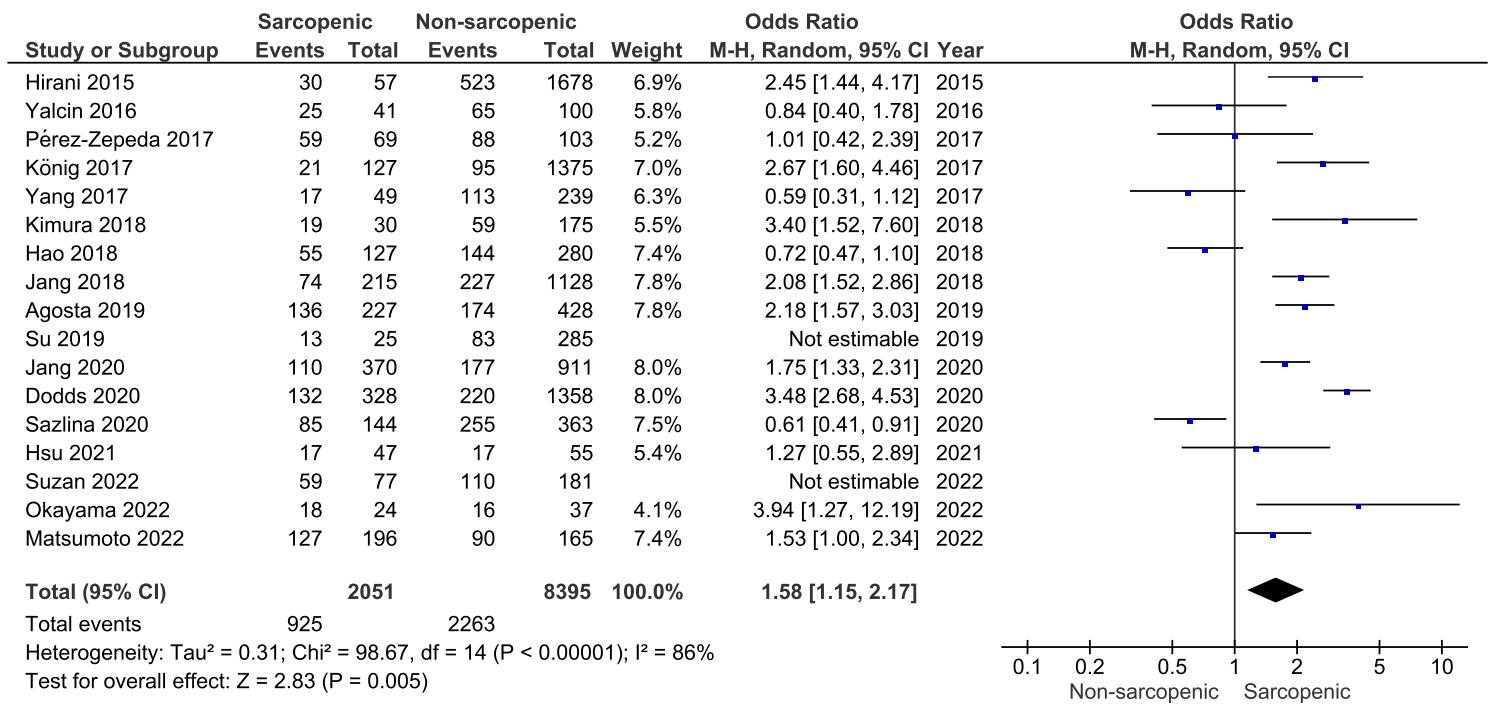

Supplement: Supplementary file 11 — Figure S11. Supporting information [file JCSM-14-671-s027.pdf]

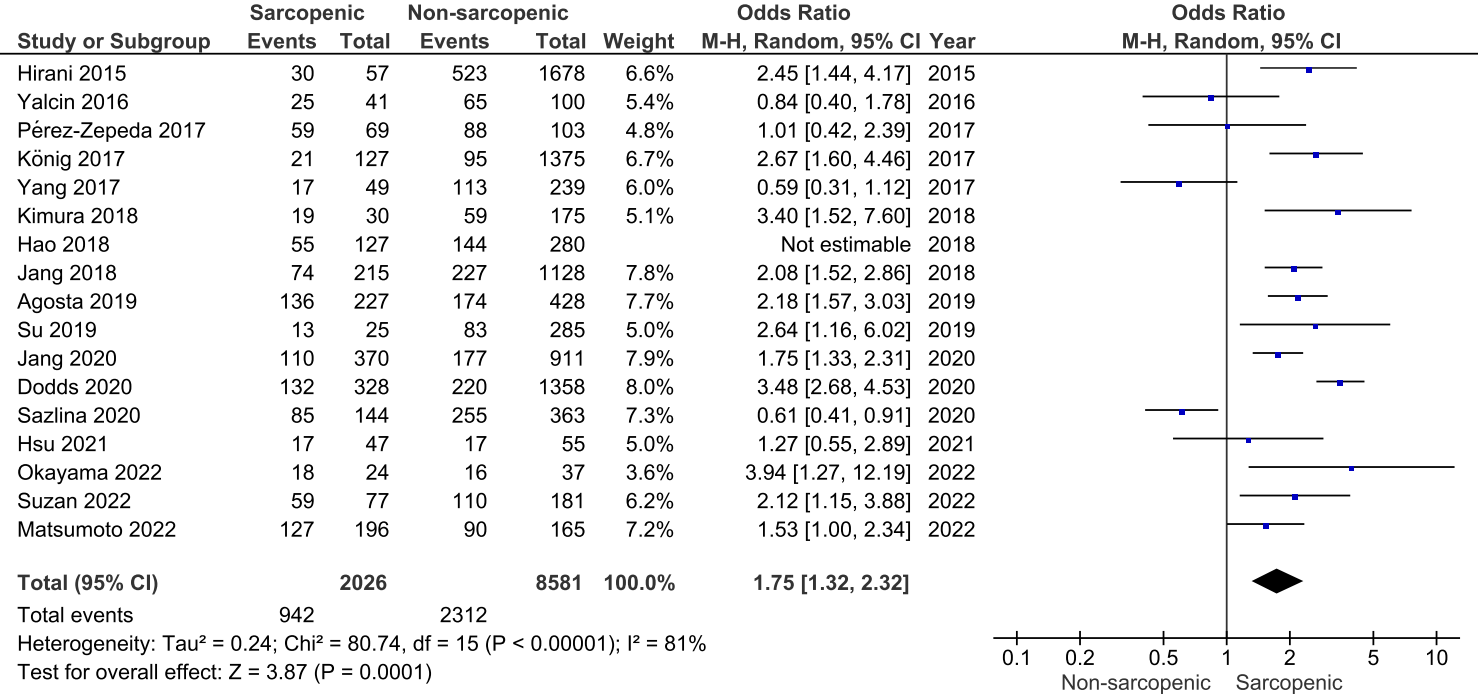

Supplement: Supplementary file 12 — Figure S12. Supporting information [file JCSM-14-671-s017.pdf]

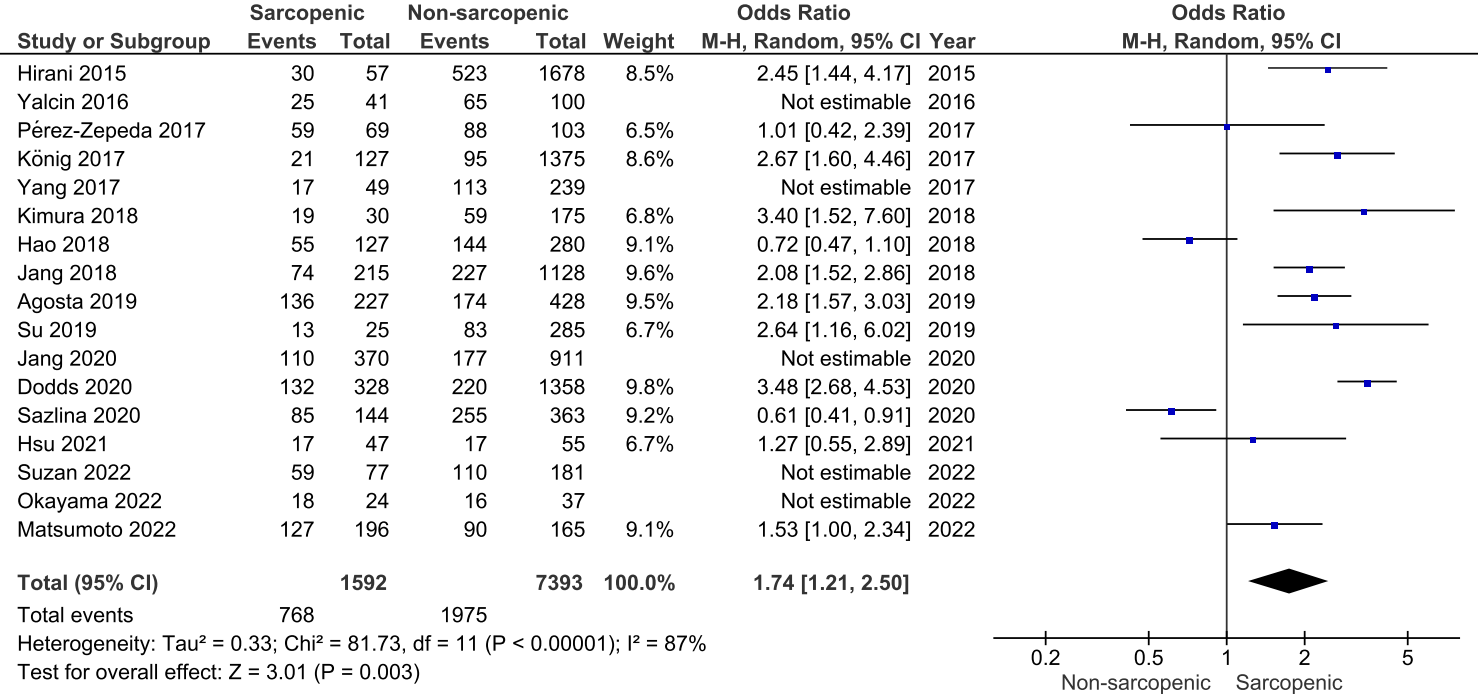

Supplement: Supplementary file 13 — Figure S13. Supporting information [file JCSM-14-671-s008.pdf]

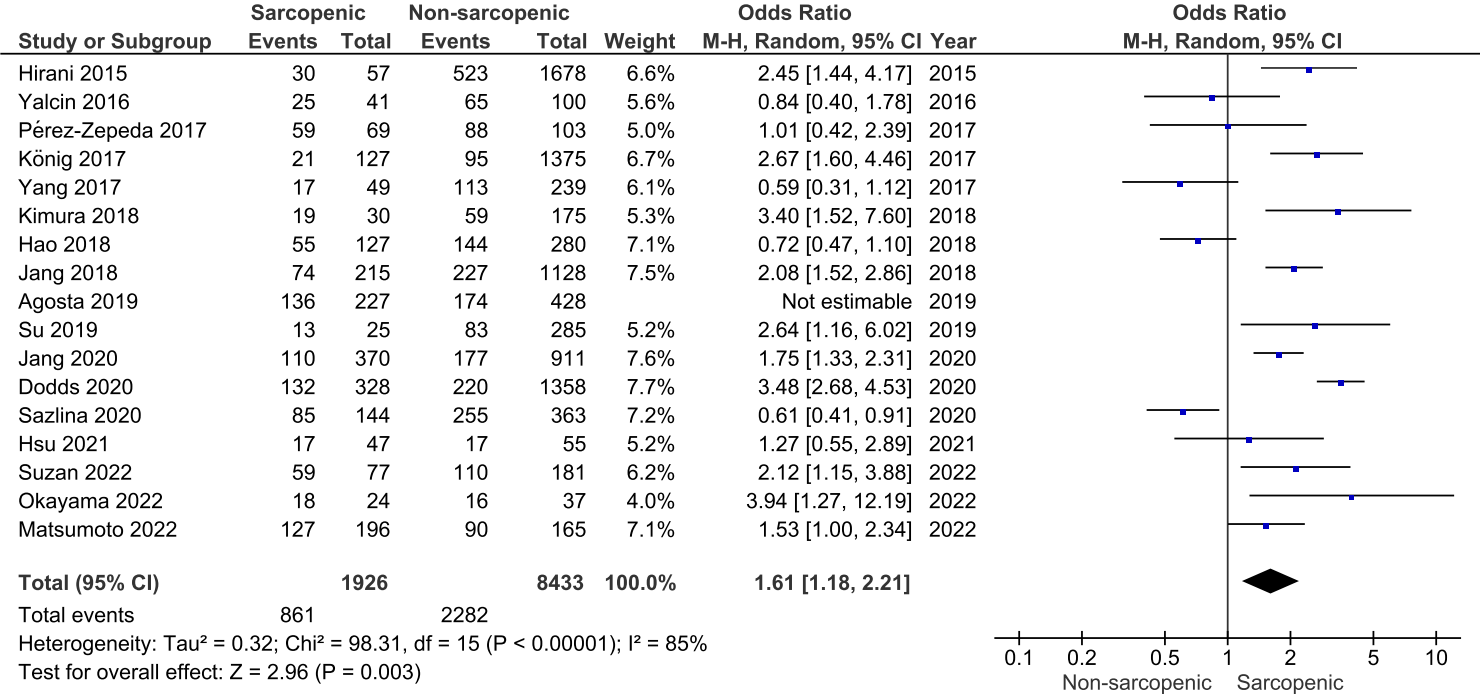

Supplement: Supplementary file 14 — Figure S14. Supporting information [file JCSM-14-671-s002.pdf]

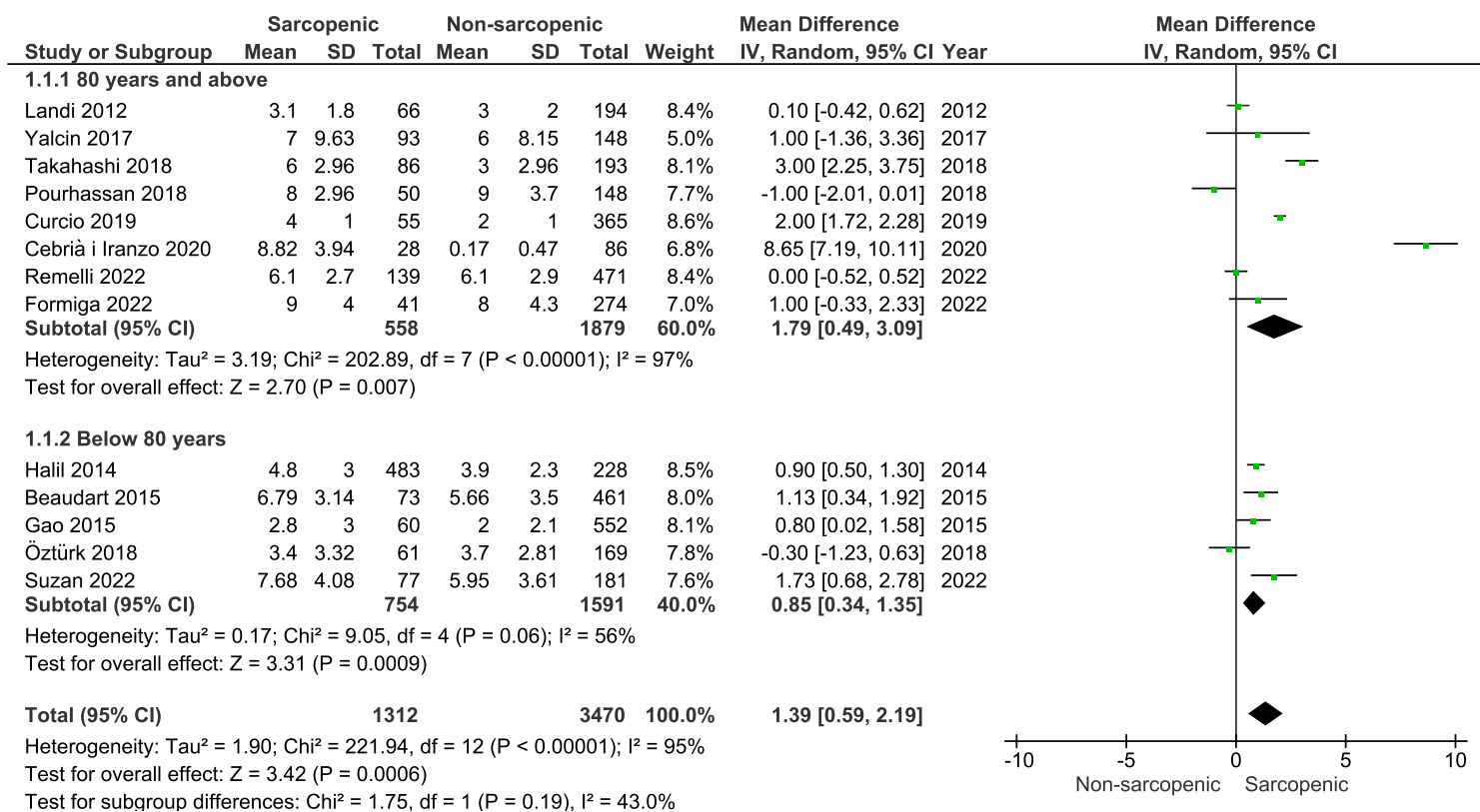

Supplement: Supplementary file 15 — Figure S15. Supporting information [file JCSM-14-671-s023.pdf]

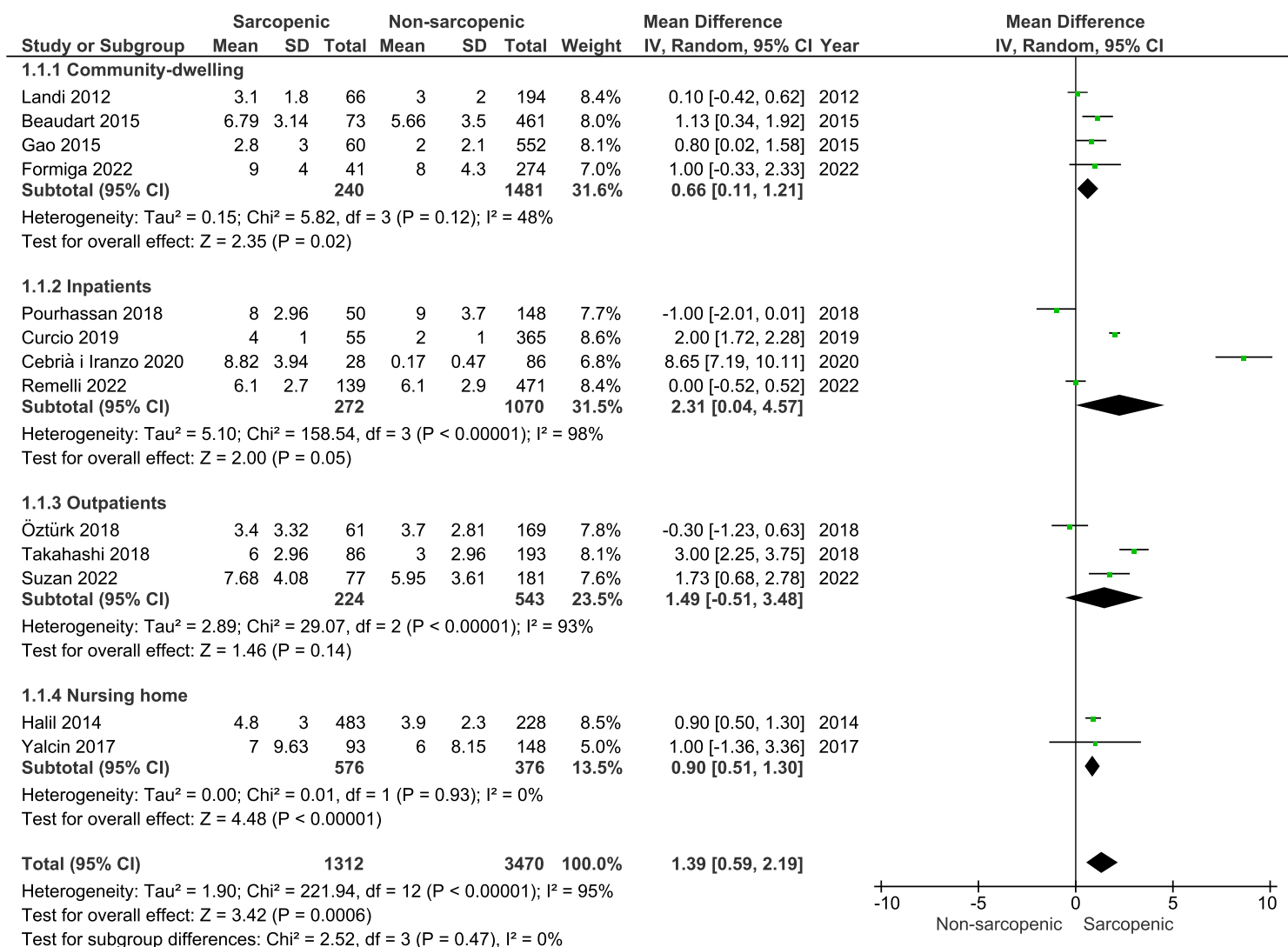

Supplement: Supplementary file 17 — Figure S17. Supporting information [file JCSM-14-671-s005.pdf]

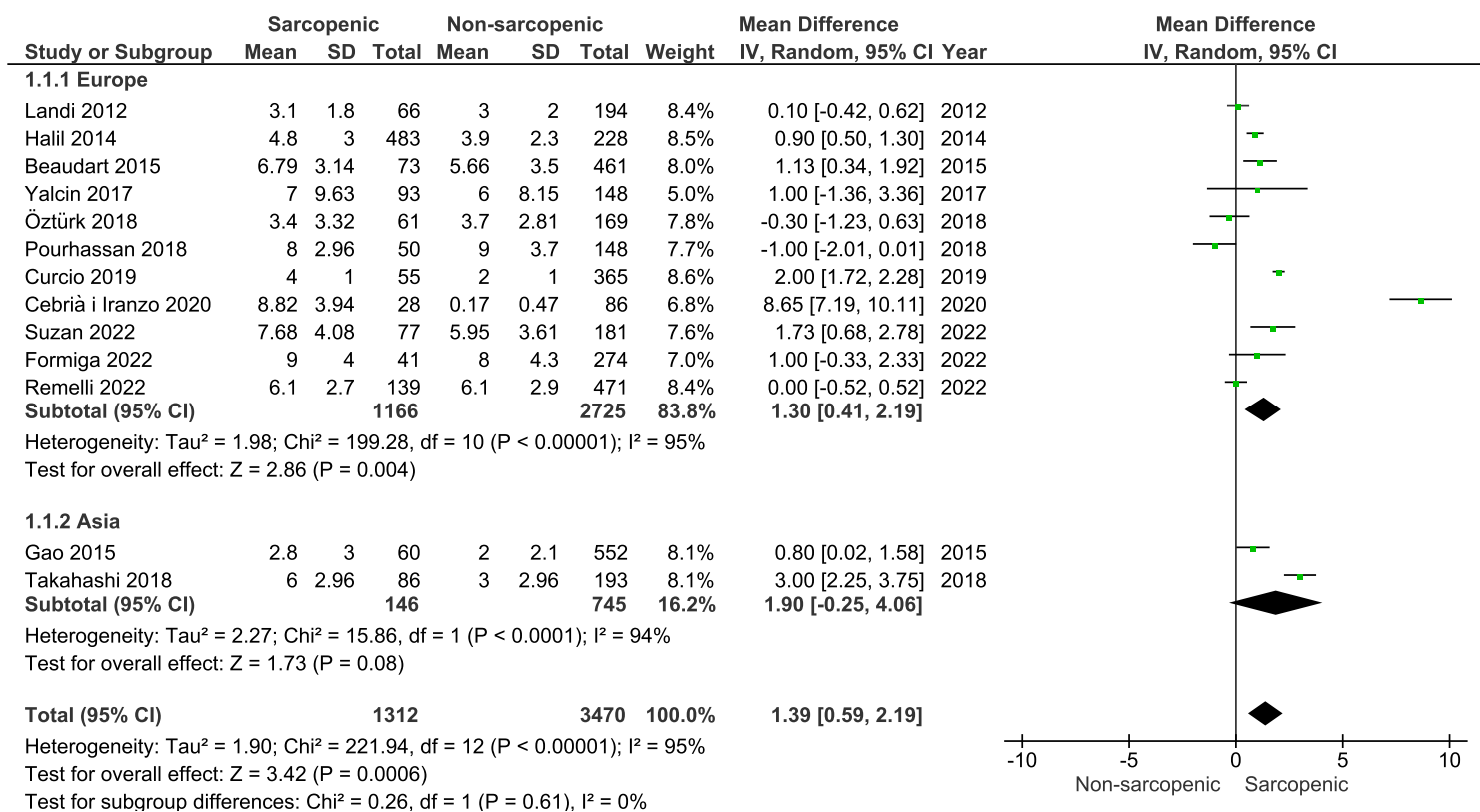

Supplement: Supplementary file 18 — Figure S18. Supporting information [file JCSM-14-671-s007.pdf]

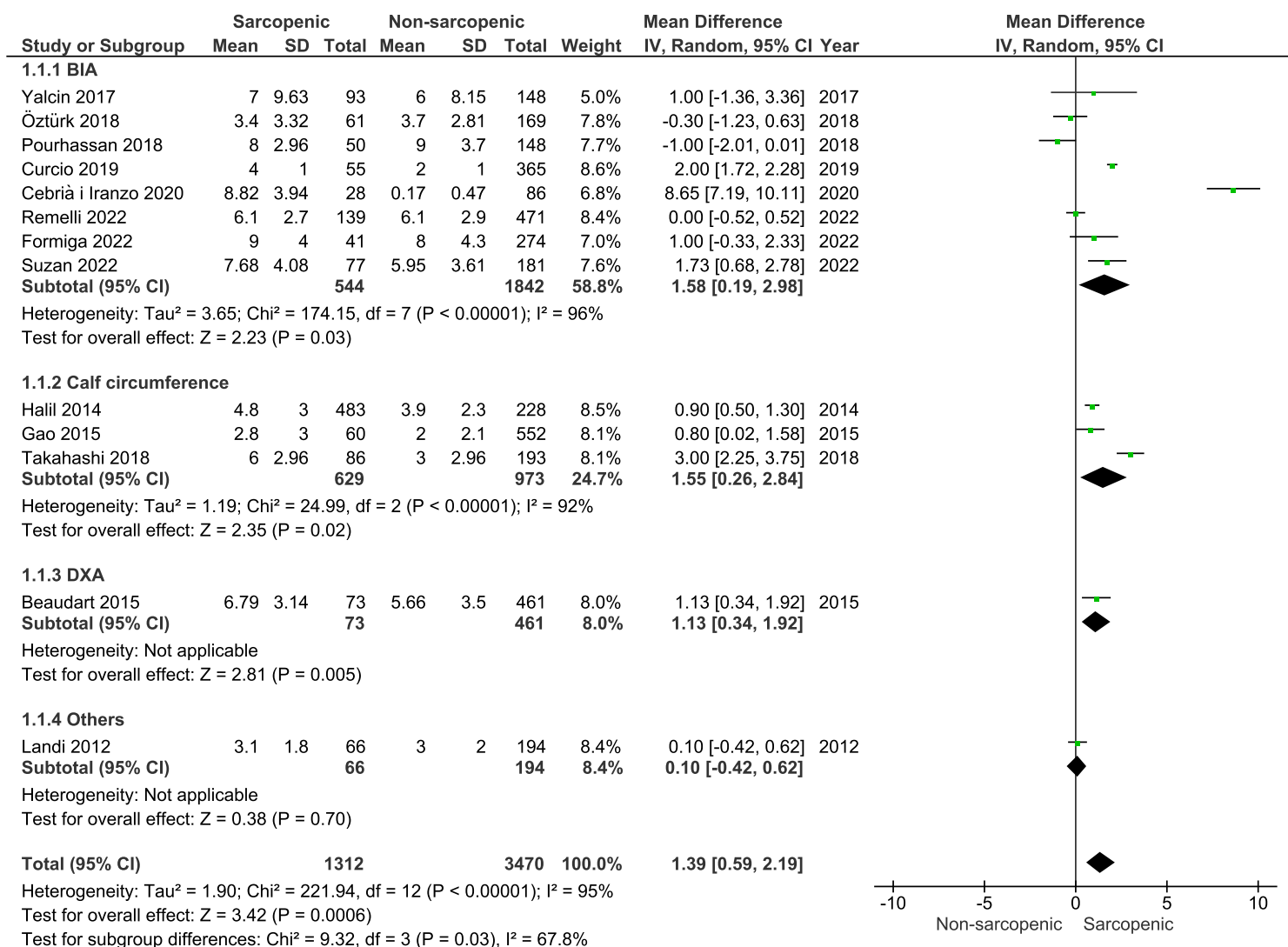

Supplement: Supplementary file 19 — Figure S19. Supporting information [file JCSM-14-671-s019.pdf]

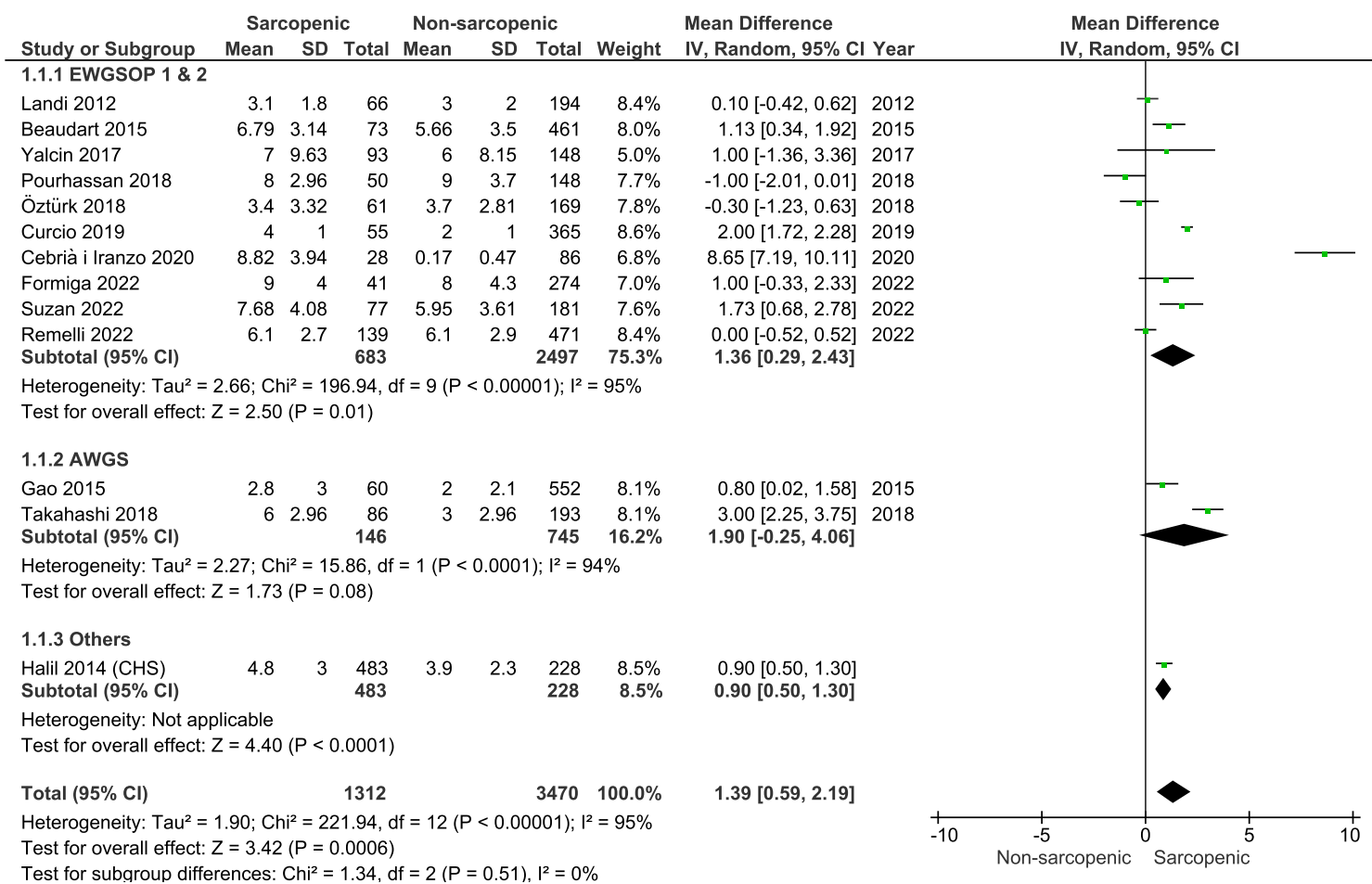

Supplement: Supplementary file 20 — Figure S20. Supporting information [file JCSM-14-671-s020.pdf]

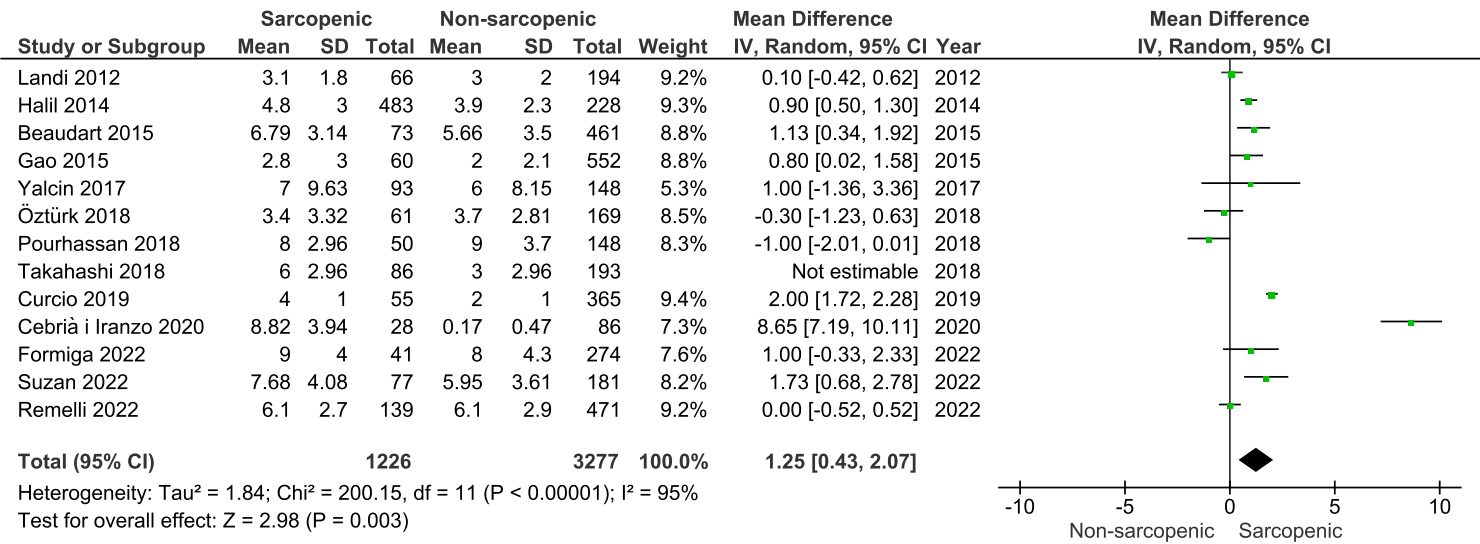

Supplement: Supplementary file 21 — Figure S21. Supporting information [file JCSM-14-671-s006.pdf]

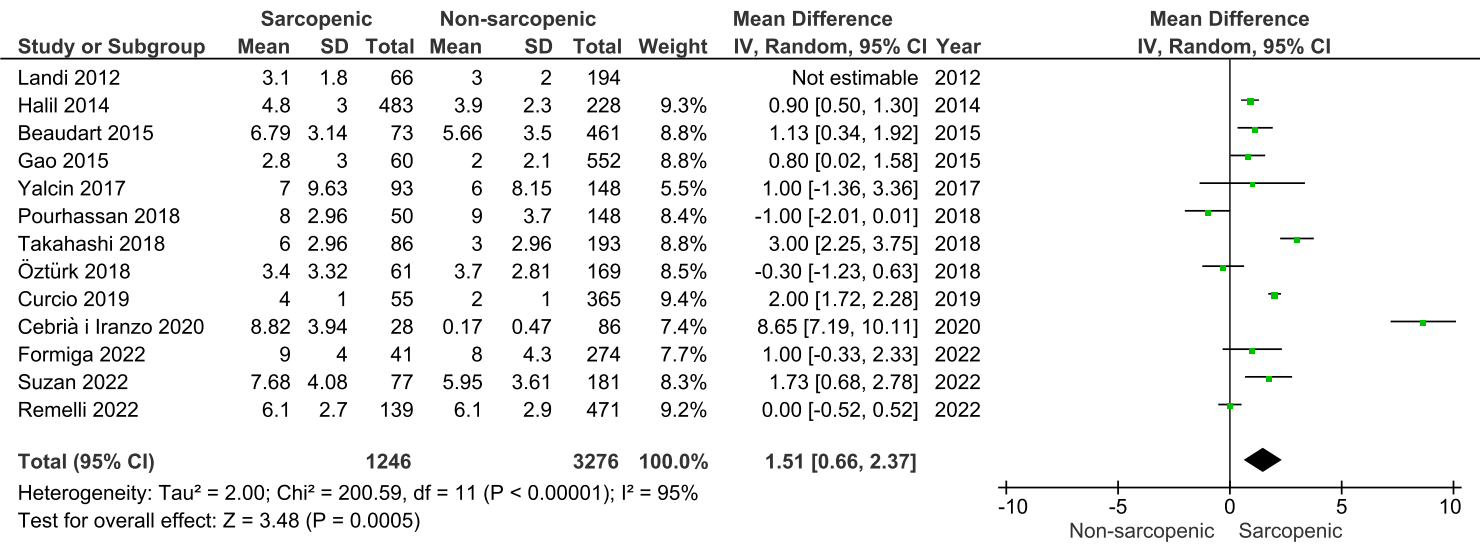

Supplement: Supplementary file 22 — Figure S22. Supporting information [file JCSM-14-671-s011.pdf]

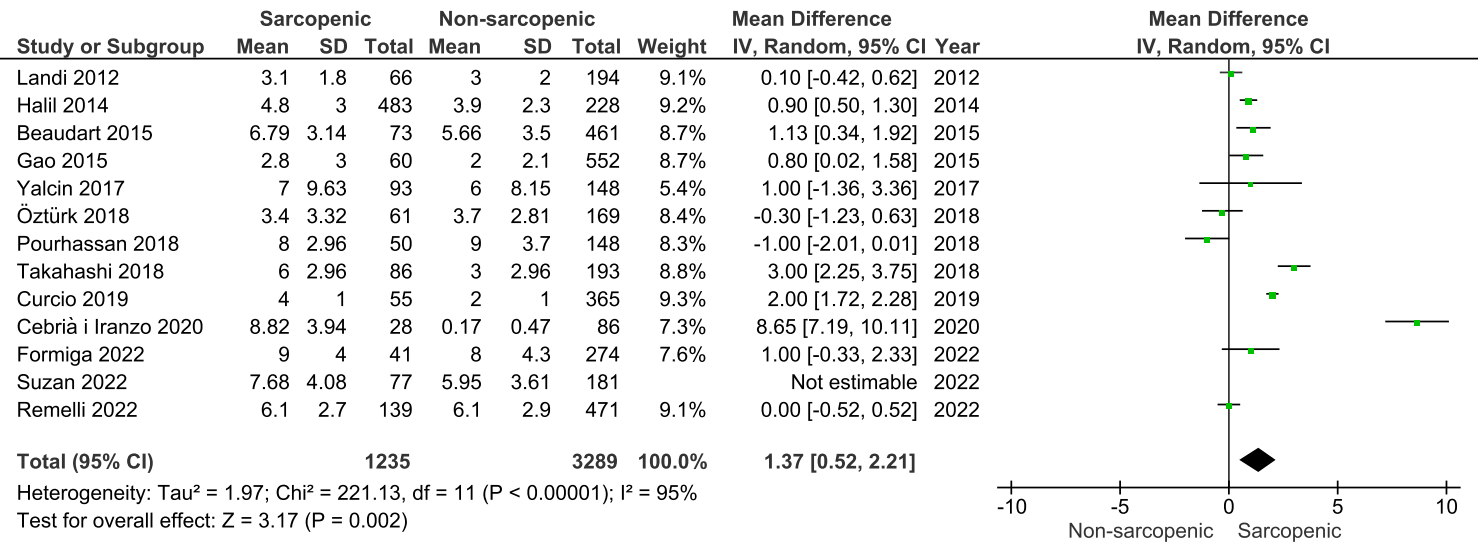

Supplement: Supplementary file 23 — Figure S23. Supporting information [file JCSM-14-671-s024.pdf]

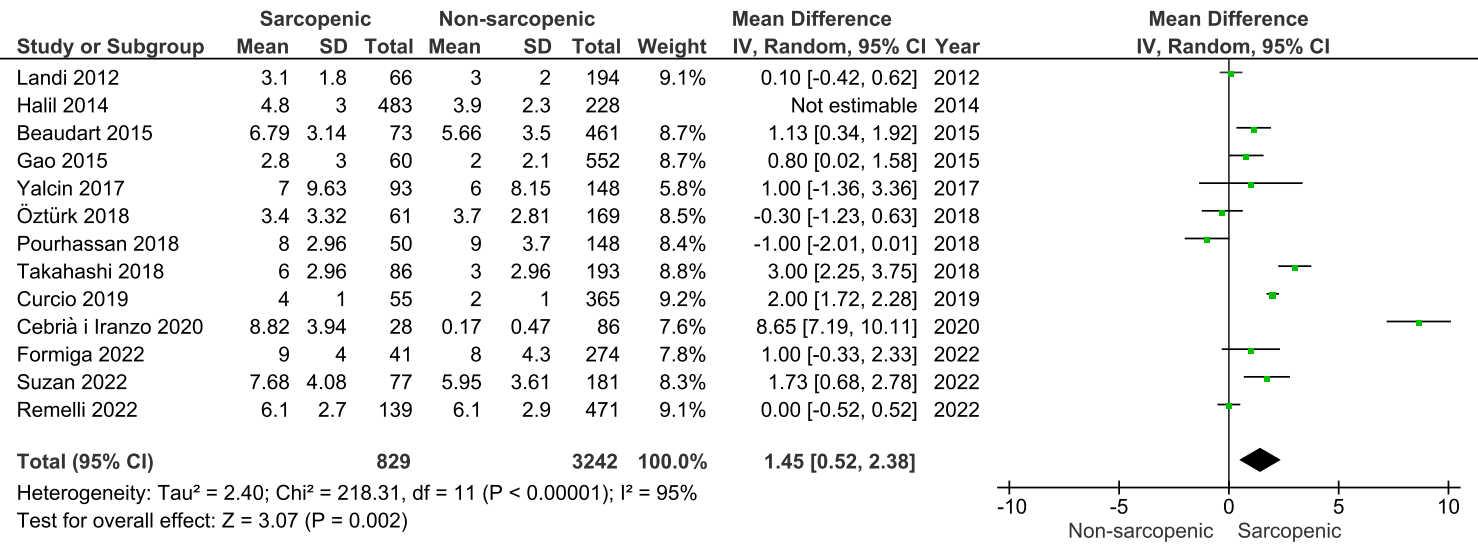

Supplement: Supplementary file 24 — Figure S24. Supporting information [file JCSM-14-671-s016.pdf]

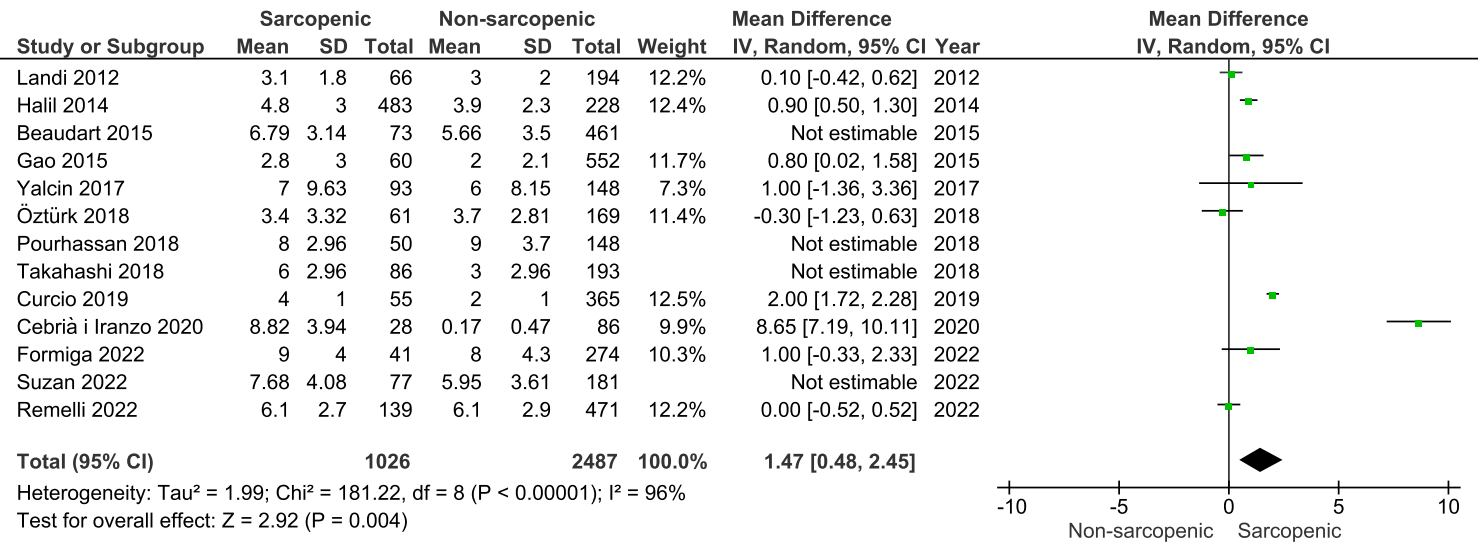

Supplement: Supplementary file 25 — Figure S25. Supporting information [file JCSM-14-671-s009.pdf]

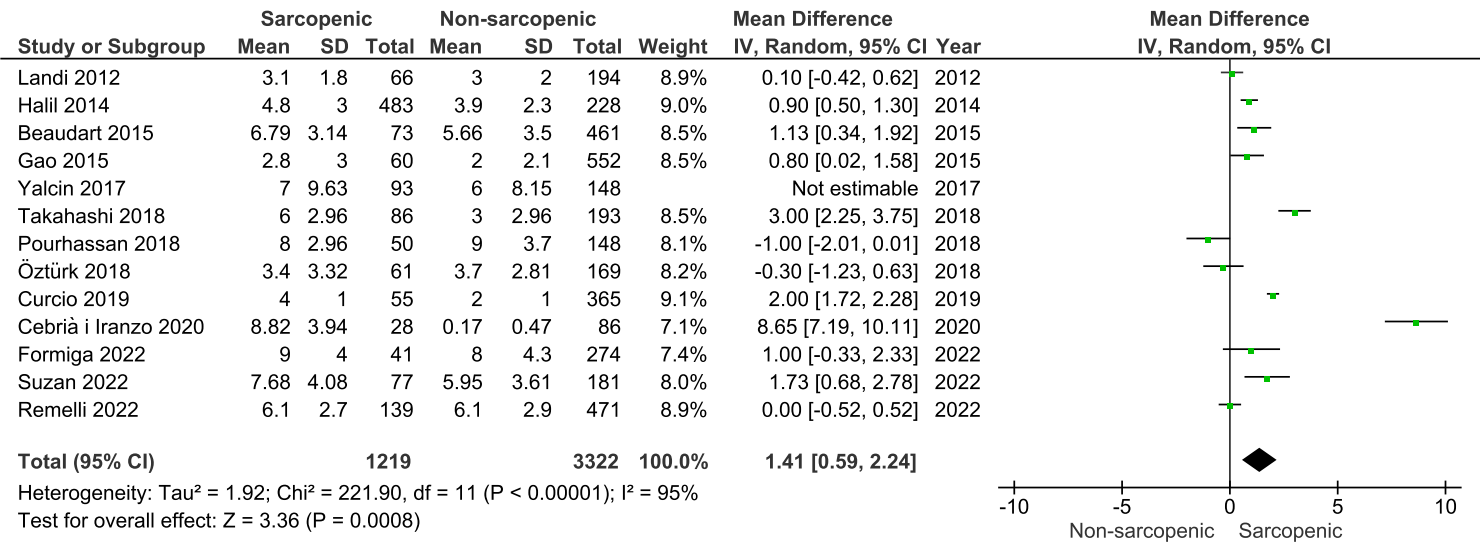

Supplement: Supplementary file 26 — Figure S26. Supporting information [file JCSM-14-671-s014.pdf]
