# Supplementary material for: Sarcopenia is associated with a greater risk of polypharmacy and number of medications: a systematic review and meta‐analysis
Source: J Cachexia Sarcopenia Muscle. 2023 Feb 13;14(2):671–83. doi: 10.1002/jcsm.13190 (PMC10067503; doi:10.1002/jcsm.13190)

	Sarcopenic			Non-sarcopenic				Mean Difference	
**Study or Subgroup**	**Mean**	**SD**	**Total**	**Mean**	**SD**	**Total**	**Weight**	**IV, Random, 95% CI**	**Year**
**1.1.1 Similar comorbidities**									
Halil 2014	4.8	3	483	3.9	2.3	228	8.5%	0.90 [0.50, 1.30]	2014
Gao 2015	2.8	3	60	2	2.1	552	8.1%	0.80 [0.02, 1.58]	2015
Pourhassan 2018	8	2.96	50	9	3.7	148	7.7%	-1.00 [-2.01, 0.01]	2018
Curcio 2019	4	1	55	2	1	365	8.6%	2.00 [1.72, 2.28]	2019
Remelli 2022	6.1	2.7	139	6.1	2.9	471	8.4%	0.00 [-0.52, 0.52]	2022
**Subtotal (95% CI)**			**787**			**1764**	**41.3%**	**0.60 [-0.35, 1.55]**	
Heterogeneity: Tau² = 1.07; Chi² = 72.21, df = 4 (P < 0.00001); I² = 94%									
Test for overall effect: Z = 1.23 (P = 0.22)									
**1.1.2 Others**									
Landi 2012	3.1	1.8	66	3	2	194	8.4%	0.10 [-0.42, 0.62]	2012
Beaudart 2015	6.79	3.14	73	5.66	3.5	461	8.0%	1.13 [0.34, 1.92]	2015
Yalcin 2017	7	9.63	93	6	8.15	148	5.0%	1.00 [-1.36, 3.36]	2017
Takahashi 2018	6	2.96	86	3	2.96	193	8.1%	3.00 [2.25, 3.75]	2018
Öztürk 2018	3.4	3.32	61	3.7	2.81	169	7.8%	-0.30 [-1.23, 0.63]	2018
Cebrià i Iranzo 2020	8.82	3.94	28	0.17	0.47	86	6.8%	8.65 [7.19, 10.11]	2020
Suzan 2022	7.68	4.08	77	5.95	3.61	181	7.6%	1.73 [0.68, 2.78]	2022
Formiga 2022	9	4	41	8	4.3	274	7.0%	1.00 [-0.33, 2.33]	2022
**Subtotal (95% CI)**			**525**			**1706**	**58.7%**	**2.01 [0.47, 3.54]**	
Heterogeneity: Tau² = 4.52; Chi² = 149.61, df = 7 (P < 0.00001); I² = 95%									
Test for overall effect: Z = 2.57 (P = 0.01)									
**Total (95% CI)**			**1312**			**3470**	**100.0%**	**1.39 [0.59, 2.19]**	
Heterogeneity: Tau² = 1.90; Chi² = 221.94, df = 12 (P < 0.00001); I² = 95%									
Test for overall effect: Z = 3.42 (P = 0.0006)									
Test for subgroup differences: Chi² = 2.35, df = 1 (P = 0.13), I² = 57.4%									

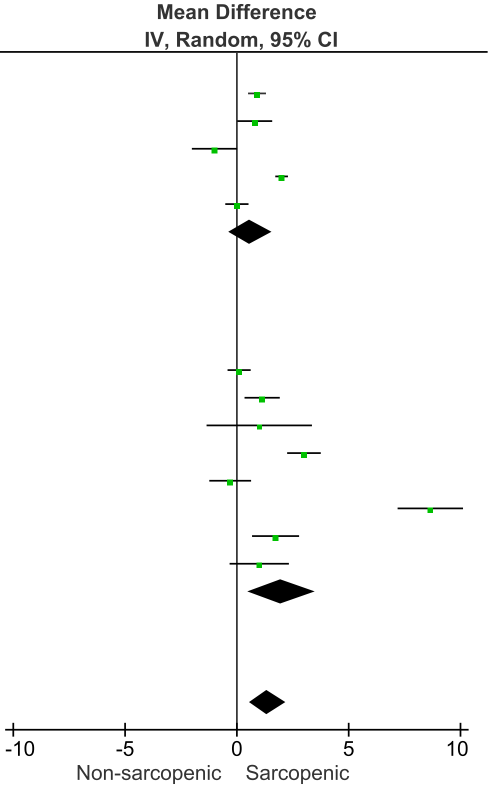

Supplement: Supplementary file 16 — Figure S16. Supporting information [file JCSM-14-671-s022.pdf]
