# Supplementary material for: Sarcopenia is associated with a greater risk of polypharmacy and number of medications: a systematic review and meta‐analysis
Source: J Cachexia Sarcopenia Muscle. 2023 Feb 13;14(2):671–83. doi: 10.1002/jcsm.13190 (PMC10067503; doi:10.1002/jcsm.13190)
Supplement: Supplementary file 27 — Table S1. Supporting information [file JCSM-14-671-s029.docx]

**Table S1.**Search terms employed in the screening based on title, abstract, and keywords in the literature search.

| **Database** | **Search terms** |
| --- | --- |
|  |  |
| PubMed | ("polypharmacy" OR “prescription*” OR “number of prescriptions” OR “multiple prescriptions”  OR “drug*” OR “numbers of drugs” OR "multiple drugs" OR “medication*” OR "multiple medications"  AND "sarcopeni*") |
| Cochrane Library | (polypharmacy OR number of prescriptions OR numbers of drugs OR number of medications OR no of medications  OR no of drugs) AND sarcopenia |
| Web of Science | TS=(((polypharmacy OR (number AND of AND prescriptions) OR (number AND of AND drugs) OR  (number AND of AND medications) OR prescriptions OR drugs OR medications) AND sarcopenia)) |
| Scopus | TITLE-ABS-KEY ((polypharmacy OR (number AND of AND prescriptions) OR (number AND of AND drugs)  OR (number AND of AND medications) OR prescriptions OR drugs OR medications) AND sarcopenia)) |
