# Supplementary material for: Sarcopenia is associated with a greater risk of polypharmacy and number of medications: a systematic review and meta‐analysis
Source: J Cachexia Sarcopenia Muscle. 2023 Feb 13;14(2):671–83. doi: 10.1002/jcsm.13190 (PMC10067503; doi:10.1002/jcsm.13190)
Supplement: Supplementary file 28 — Table S2 and Table S3. Supporting information [file JCSM-14-671-s018.docx]

**Table S2.** Subgroup analyses for studies evaluating the impact of polypharmacy on subjects with and without sarcopenia.

|  | Subgroup | N^o^ of studies | Odds ratio (95% CI) | P-value | I^2^ |
| --- | --- | --- | --- | --- | --- |
| Age | < 80 years  ≥ 80 years | *k* = 9  *k* = 8 | 2.02 (1.36, 3.01)  1.27 (0.82, 1.97) | P < 0.01  P = 0.28 | 86%  80% |
| Geographical location | Europe  Asia | *k* = 5  *k* = 10 | 2.23 (1.52, 3.27)  1.42 (0.97, 2.07) | P < 0.01  P = 0.07 | 73%  83% |
| Similar  health  status | Similar comorbidities | *k* = 4 | 1.38 (0.74, 2.56) | P = 0.31 | 57% |
| Population | Community-dwelling  Inpatients  Outpatients | *k* = 9  *k* = 5  *k* = 2 | 2.00 (1.35, 2.97)  1.11 (0.65, 1.89)  2.51 (1.55, 4.07) | P < 0.01  P = 0.70  P < 0.01 | 86%  83%  0% |
| Polypharmacy definition | 5 or more medications | *k* = 15 | 1.63 (1.20, 2.21) | P < 0.01 | 85% |
| Sarcopenia definition | EWGSOP 1 & 2  AWGS | *k* = 7  *k* = 8 | 1.95 (1.38, 2.76)  1.30 (0.81, 2.08) | P < 0.01  P = 0.28 | 75%  84% |
| Muscle mass assessment tool | BIA  DXA | *k* = 12  *k* = 3 | 1.54 (1.13, 2.11)  2.25 (1.55, 3.27) | P < 0.01  P < 0.01 | 78%  17% |

**Table S3.** Subgroup analyses for studies evaluating the differences on subjects with and without sarcopenia regarding the number of medications.

|  | Subgroup | N^o^ of studies | Mean Difference (95% CI) | P-value | I^2^ |
| --- | --- | --- | --- | --- | --- |
| Age | < 80 years  ≥ 80 years | *k* = 5  *k* = 9 | 0.85 (0.34, 1.35)  1.79 (0.49, 3.09) | P < 0.01  P < 0.01 | 56%  97% |
| Geographical location | Europe  Asia | *k* = 11  *k* = 2 | 1.30 (0.41, 2.19)  1.90 (-0.25, 4.06) | P < 0.01  P = 0.08 | 95%  94% |
| Similar  health  status | Similar comorbidities | *k* = 5 | 0.60 (-0.35, 1.55) | P = 0.22 | 94% |
| Population | Community-dwelling  Inpatients  Nursing home  Outpatients | *k* = 4  *k* = 4  *k* = 2  *k* = 3 | 0.66 (0.11, 1.21)  2.31 (0.04, 4.57)  0.90 (0.51, 1.30)  1.49 (-0.51, 3.48) | P = 0.02  P = 0.05  P < 0.01  P = 0.14 | 48%  98%  0%  93% |
| Sarcopenia definition | EWGSOP  1 & 2  AWGS | *k* = 11  *k* = 2 | 1.36 (0.29, 2.43)  1.90 (-0.25, 4.06) | P = 0.01  P = 0.08 | 95%  94% |
| Muscle mass assessment tool | BIA  Calf circumference | *k* = 8  *k* = 3 | 1.58 (0.19, 2.98)  1.55 (0.26, 2.84) | P = 0.03  P = 0.02 | 96%  92% |
