# Supplementary material for: Sarcopenia is associated with a greater risk of polypharmacy and number of medications: a systematic review and meta‐analysis
Source: J Cachexia Sarcopenia Muscle. 2023 Feb 13;14(2):671–83. doi: 10.1002/jcsm.13190 (PMC10067503; doi:10.1002/jcsm.13190)
Supplement: Supplementary file 29 — Table S4. Supporting information [file JCSM-14-671-s004.docx]

**Table S4.** Quality assessment of the included studies based on the Methodological Index for Non-Randomized Studies (MINORS) tool.

|  |  |  |  |  |  |  |  |  |  |  |
| --- | --- | --- | --- | --- | --- | --- | --- | --- | --- | --- |
| Author, Year | **Aim** | **Inclusion of consecutive patients** | **Prospective collection of data** | **Endpoints appropriate to the aim of the study** | **Unbiased assessment of the study endpoint** | **Follow-up period appropriate to the aim of the study** | **Loss to follow up less than 5%** | **Prospective calculation of the study size** | **Total** | **Risk of bias** |
| Formiga, 2022 | 2 | 2 | 1 | 2 | 2 | 2 | 0 | 0 | 11/16 | Low |
| Matsumoto, 2022 | 1 | 2 | 1 | 2 | 2 | 2 | 2 | 2 | 14/16 | Low |
| Okayama, 2022 | 2 | 1 | 1 | 1 | 2 | 2 | 0 | 0 | 9/16 | Some concerns |
| Remelli, 2022 | 2 | 2 | 0 | 2 | 2 | 2 | 0 | 0 | 10/16 | Low |
| Suzan, 2022 | 2 | 2 | 1 | 2 | 2 | 2 | 0 | 0 | 11/16 | Low |
| Hsu, 2021 | 2 | 2 | 0 | 2 | 2 | 2 | 2 | 0 | 12/16 | Low |
| Cebrià i Iranzo, 2020 | 2 | 2 | 1 | 2 | 2 | 2 | 0 | 2 | 11/16 | Low |
| Dodds, 2020 | 2 | 2 | 1 | 2 | 2 | 2 | 0 | 0 | 11/16 | Low |
| Jang, 2020 | 1 | 2 | 0 | 2 | 2 | 2 | 0 | 0 | 9/16 | Some concerns |
| Sazlina, 2020 | 2 | 2 | 0 | 2 | 2 | 2 | 0 | 2 | 12/16 | Low |
| Agosta, 2019 | 1 | 1 | 0 | 2 | 2 | 0 | 0 | 0 | 6/16 | High |
| Curcio, 2019 | 1 | 2 | 1 | 2 | 2 | 2 | 2 | 2 | 14/16 | Low |
| Su, 2019 | 2 | 2 | 0 | 2 | 2 | 2 | 0 | 2 | 12/16 | Low |
| Hao, 2018 | 2 | 2 | 1 | 2 | 2 | 0 | 0 | 0 | 11/16 | Low |
| Jang, 2018 | 1 | 2 | 2 | 2 | 2 | 2 | 2 | 0 | 13/16 | Low |
| Kimura, 2018 | 2 | 0 | 0 | 2 | 2 | 2 | 2 | 0 | 10/16 | Low |
| König, 2018 | 2 | 2 | 1 | 2 | 2 | 2 | 0 | 0 | 11/16 | Low |
| Öztürk, 2018 | 1 | 2 | 1 | 2 | 2 | 2 | 0 | 0 | 10/16 | Low |
| Pourhassan, 2018 | 2 | 2 | 2 | 2 | 2 | 0 | 0 | 0 | 10/16 | Low |
| Takahashi, 2018 | 1 | 2 | 2 | 2 | 1 | 2 | 2 | 0 | 12/16 | Low |
| Pérez-Zepeda,2017 | 2 | 2 | 2 | 2 | 2 | 2 | 2 | 0 | 14/16 | Low |
| Yalcin, 2017 | 1 | 2 | 0 | 2 | 1 | 1 | 0 | 0 | 7/16 | High |
| Yang, 2017 | 2 | 2 | 1 | 2 | 2 | 0 | 0 | 0 | 9/16 | Some concerns |
| Yalcin, 2016 | 1 | 2 | 2 | 2 | 1 | 2 | 2 | 0 | 12/16 | Low |
| Beaudart, 2015 | 2 | 2 | 2 | 2 | 2 | 2 | 2 | 2 | 16/16 | Low |
| Gao, 2015 | 2 | 2 | 2 | 2 | 2 | 2 | 2 | 0 | 14/16 | Low |
| Hirani, 2015 | 2 | 2 | 1 | 1 | 2 | 2 | 2 | 0 | 14/16 | Low |
| Halil, 2014 | 2 | 2 | 1 | 2 | 2 | 2 | 2 | 0 | 13/16 | Low |
| Landi, 2012 | 2 | 2 | 1 | 2 | 2 | 2 | 2 | 0 | 13/16 | Low |
